# Effects of Friendship Influence and Normative Pressure on Adolescent Attitudes and Behaviors

**DOI:** 10.1177/01461672251366388

**Published:** 2025-09-25

**Authors:** Tibor Zingora, Andreas Flache

**Affiliations:** 1 University of Groningen, Netherlands; 2 Czech Academy of Sciences, Czechia

**Keywords:** social network, group norms, social influence, friendship, school

## Abstract

Peers are assumed to be crucial in shaping adolescents’ attitudes and behaviors in schools; what peers yield social influence over adolescents is less clear. We disentangled two mechanisms of peer influence: friendship influence and normative pressure. Friendship influence refers to the impact of friends’ attitudes and behaviors, while normative pressure stems from the influence of peers from the same social group, such as gender group. We adopted a network perspective, recognizing that peer influence can be shaped by changing relationships among peers. We investigated friendship influence and normative pressure based on sharing the same gender and ethnicity in shaping academic drive, prejudice, and religiosity. We analyzed a sample of German school grades (*N* = 2,838; 29 school grades) using stochastic actor-oriented modeling. Adolescents influenced each other via both friendship influence and normative pressure. These findings improve our understanding of who influences whom in schools and open new research avenues.

Peer influence in schools is a pervasive force in the lives of adolescents, impacting their attitudes and behaviors. Knowing who influences whom in schools is crucial for mapping and intervening in the development of various outcomes among adolescents (Veenstra & Laninga-Wijnen, 2022). Here, we combine research on social influence in schools, group processes, and social network science to separate and test two different forms of peer influence: friendship influence and group-based normative pressure. Knowing if peer influence is based on both friendship influence and normative pressure, or only one of them, will enhance the general understanding of peer influence within schools, provide critical implications for school interventions, and improve predictions of the development of adolescents’ attitudes and behaviors. To this end, we investigated how friendship influence and normative pressure shaped the development of academic drive, prejudice, and religiosity among adolescents, aiming to disentangle their unique contributions to peer influence and potentially simultaneous effects.

In a school environment, adolescents are susceptible to peer influence, especially to that of friends (Giletta et al., 2021). Friendship is a personal relationship defined by liking, intimacy, and trust, which makes it more likely that adolescents will be influenced by the attitudes and behaviors of their friends compared to random peers (Berndt, 1996; Levine & Wheeless, 1997). Friendship is also characterized by frequent interactions, granting more frequent and intense opportunities for friends to influence each other (Bandura, 1977; Paluck & Shepherd, 2012). In this vein, numerous studies attest to the profound impacts of friendship influence on the development of attitudes and behaviors among adolescents (Bracegirdle et al., 2022; DeLay et al., 2022; Geven et al., 2013; Gremmen et al., 2019; Li et al., 2023; Steglich et al., 2006; Weerman, 2011; Zingora et al., 2019). Friendships thus represent crucial channels for spreading attitudes and behaviors among adolescents.

However, in the school setting, the flow of influence is not necessarily limited to friendships. Adolescents can also observe the attitudes and behaviors of peers who are not their friends and potentially be influenced by them. This mechanism of influence, known as normative pressure, is based on affiliation to the same group (such as gender or ethnic group) and the pressure to conform to group norms (Hogg & Reid, 2006). Attitudes and behaviors of peers who belong to the same group can be understood as cues about acceptable behavior, and adolescents should thus be inclined to change their attitudes and behavior in the direction of peers’ attitudes and behaviors (Cialdini et al., 1991). Normative pressure and friendship influence are compatible but separate types of peer influence. They can shape the spread of attitudes and behaviors in schools independently; thus, peers could influence each other because they are friends as well as due to belonging to the same group.

Are we missing a crucial part of social influence among adolescents if we focus exclusively on friendship influence or normative pressure without considering their co-occurrence and their interplay? Social network studies mostly limit direct peer influence to interpersonal relationships, such as friendships (e.g., Steglich et al., 2010). Consequently, adolescents have been assumed to be influenced only by peers with whom they formed interpersonal relationships, potentially neglecting a considerable part of the social influences to which they are exposed in school. Friendship and group membership have distinct qualities (Newcomb & Bagwell, 1995; Tajfel et al., 1979), indicating different types of peer influence. Theoretical research on norm enforcement even points to a “weakness of strong ties” (Flache, 2002; Flache & Macy, 1996), like friendships, suggesting that adolescents may refrain from enforcing their friends’ compliance with group norms to avoid the loss of other valuable benefits in the friendship. To avoid conflict with a friend, friends can avoid enforcing norms upon each other. However, ingroup members, including friends who belong to the same group, can be a passive source of normative pressure when normative attitudes and behaviors are observed (Cialdini et al., 1991). Although various theories suggest the existence of normative pressure (Cialdini et al., 1991; Hogg & Reid, 2006; Tajfel et al., 1979; Turner et al., 1987), it should not be yielded only by friends, if attitudes and behaviors of ingroup peers are accessible and can be observed. This indicates that normative pressure and friendship influence are distinct forms of peer influence with unique consequences for the development of adolescents’ attitudes and behaviors.

## Distinguishing Friendship Influence and Normative Pressure

**Figure 1.**
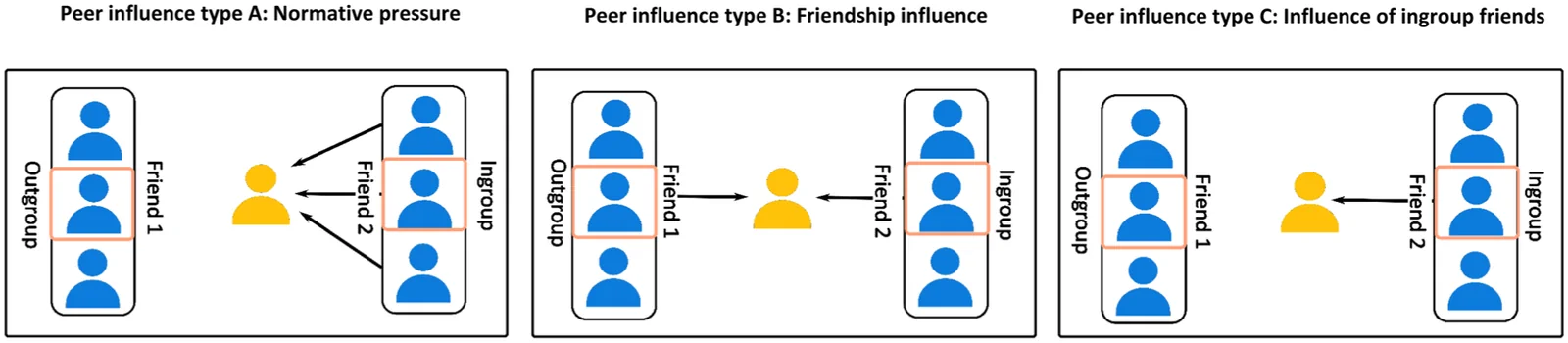
Potential types of peer influence. This figure presents three investigated types of peer influence. In some studies, peer influence was restricted to friends, omitting the normative pressure of peers who were not labeled as friends (Van Zalk et al., 2013; Zingora et al., 2019). Some studies even found that only ingroup friends influence adolescents (Bracegirdle et al., 2022). These studies were guided by the assumption that ingroup friends may be the source of normative pressure from the ingroup to an adolescent, granting ingroup friends an especially large influence over adolescents’ attitudes and behaviors. This would mean that studying the influence of ingroup friends could substitute for the investigation of normative pressure. On the other hand, adolescents have frequent opportunities to observe peers in schools, and they might not rely on their ingroup friends to communicate group norms (Flache, 2002; Flache & Macy, 1996). Therefore, studies that reduce peer influence to influence from ingroup friends could actually miss an important part of the picture: normative pressure from all peers who belong to the same social group. The validity of the assumption that ingroup friends are the sole source of normative pressure is thus unclear.

The theory implies that friendship influence and normative pressure are two distinct types of peer influence (for a graphic depiction of all investigated types of peer influence, see Figure 1). Friendships are distinct from other peer relationships in that they are chosen by adolescents and characterized by features like intimacy, affection, cooperation, conflict resolution, and a reduced emphasis on dominance (Newcomb & Bagwell, 1995). These unique characteristics create opportunities for open communication and persuasive exchanges between individuals. In contrast, peers belonging to the same social group share certain characteristics, such as gender or ethnicity, but do not necessarily need to be friends or even like each other. Nevertheless, group membership can facilitate the adoption of shared norms and behaviors (Tajfel et al., 1979; Turner et al., 1987). People, in general, perceive the typical behavior in their group, which can lead to a change in their behavior (Cialdini et al., 1991, 2006).

Recent empirical research further supports the notion that normative peer pressure influences adolescents beyond friendship influence. One study distinguished the influence of all girls from friendship influence on girls’ preference for STEM subjects (Raabe et al., 2019). All students were subjected to friendship influence, and girls tended to retain their STEM preferences if other girls in their classroom liked STEM. The critical questions remain unanswered, though: Could normative pressure be neglected when influence from ingroup friends is considered? Does this hold across a variety of outcomes? Is the overall pressure to comply with peer group norms severely underestimated when only friendship influence is considered? More specifically, we ask what the distinct and combined effects are of friendship influence and normative pressure across various attitudes and behaviors (i.e., academic drive, prejudice, religiosity), and how friendship influence and normative pressure operate in a complex school environment characterized by overlapping social groups (i.e., ethnicity and gender). Moreover, friends and ingroup peers overlap, and both processes of peer influence—friendship influence and normative pressure—can operate simultaneously. An important reason to answer these questions is that these processes have potentially different implications for the development of students’ attitudes and behaviors. For example, many interventions aim to spread attitudes inconsistent with group norms, such as reduced prejudice toward outgroups. Interventions might be directed at influencing students who occupy central positions within a friendship network, assuming that these students are particularly influential in changing group norms. Yet, these students might not be as influential if we consider that students experience normative pressure not only from their friends, but also from ingroup peers not connected to them via friendships.

The theoretical model that generates our research hypotheses is portrayed in Figure 1, which illustrates two forms of peer influence we hypothesize to be distinct and their combined form. The yellow individual represents an adolescent who is potentially influenced by peers (blue). Arrows represent peer influence. Friends are labeled by orange rectangles. Outgroup peers (i.e., peers who do not belong to the same social group) and ingroup peers (i.e., peers who belong to the same social group) are in black rectangles. Peer influence type A represents normative pressure, whereby we expect that adolescents are influenced by average attitudes and behaviors among ingroup peers. Peer influence type B represents friendship influence, whereby we expect that adolescents are influenced by the average attitude or behavior among friends. Peer influence type C represents the combined effect of normative pressure and friendship influence: the influence of ingroup friends, whereby we expect that adolescents are influenced by average attitudes and behaviors among ingroup friends. All three types can affect adolescents simultaneously, but they can differ in strength and in the content of influence. For example, if the focal adolescent (yellow) is female, we would expect that this girl would be influenced by all other girls via normative pressure, by all friends via friendship influence, and uniquely by girlfriends via the influence of ingroup friends.

## Social Network Science and Peer Influence

The social network approach provides a unique perspective on the spread of attitudes and behaviors in schools and enables us to disentangle friendship influence and normative pressure while controlling for tendencies that could result in spurious links between peer influence and the development of attitudes and behaviors among students (Steglich et al., 2010). Adolescents can be connected through dyadic relationships, such as friendship, thereby creating a social network (Veenstra & Laninga-Wijnen, 2022; Wölfer & Hewstone, 2017). In this network, adolescents should influence each other along friendship lines, which means that the changing structure of a friendship network and the dynamic processes responsible for the network development determine the direction of friendship influence among classmates (Veenstra et al., 2013). Changes in adolescent friendships over time can be used to understand and control the key dynamic processes in schools that shape the formation of friendships and the development of attitudes and behaviors (e.g., Leszczensky et al., 2016; Stark, 2015). For instance, social network analysis techniques such as stochastic actor-oriented models can help determine how much of the similarity between friends’ attitudes and behaviors is due to forming friendships with similar peers and how much is due to social influence after a friendship was created (Steglich et al., 2010). A failure to account for a tendency to select friends who are similar in the very beginning could produce erroneous conclusions about friendship influence. The social network approach has benefited social psychology theories, such as the Intergroup Contact Theory (Bracegirdle et al., 2022; Zingora, 2024). The benefits of the social network approach have been employed in network interventions (Paluck et al., 2016; Valente, 2012; Veenstra & Laninga-Wijnen, 2022). Such interventions can exploit the knowledge of the social influence flow among adolescents to spread a desirable outcome among classroom members.

In some cases, the social influence flow could be restricted to the links between network members. This typically applies when network members lack alternative means of communicating specific attitudes and behaviors besides links that connect network members. Although normative pressure could be an explanation for the spread of attitudes and behaviors within classrooms, adolescents belonging to the same group would not be connected in a network, and their mutual influence would not be captured if we rely only on friendship influence. Including static relationships among adolescents, such as group membership, would thus result in a more complete and accurate network of social influence flow. The methodological innovation of disentangling friendship influence from normative pressure could be included in the social network framework and utilize established tools such as stochastic actor-oriented models. While the separation of friendship influence and normative pressure offers multiple benefits, the inclusion of our operationalization of normative pressure within network studies would be relatively simple and could be adopted more widely.

From the social network perspective, one can picture both types of peer influence as two networks. One network represents the ties between friends, while the other connects peers from the same social group. Through these networks, students influence one another, and both types of connections can jointly help to explain the diffusion of attitudes and behaviors in school settings. Friends often come from the same group as adolescents (Leszczensky & Pink, 2020; McPherson et al., 2001). This network approach enables disentangling friendship influence and normative pressure, even when friends and same-group peers partially overlap. Furthermore, this approach also allows us to investigate whether ingroup friends are particularly influential, besides estimating friendship influence and normative pressure.

Using the network approach offers additional advantages for studying normative pressure. A recent meta-analysis of longitudinal studies demonstrated a robust effect of friendship influence on adolescents’ behaviors (Giletta et al., 2021). The authors suggested that adolescents often overestimate the similarity between their attitudes or behaviors and those of their friends. Consequently, studies that relied on self-reports of others’ behaviors were excluded. Instead, the analysis emphasized studies where all students independently reported their own behavior and identified their peer connections, particularly friendships. Our social network approach offers a similar avenue to investigating friendship influence and further extends it to study normative pressure. We rely on reports from all students in a given context and use belonging to the same group as connections that enable them to influence each other.

## Implications

Developing an empirical and methodological basis for distinguishing and combining effects of friendship influence and normative pressure in social networks models of influence on adolescents’ attitudes and behaviors would have two major implications: it would improve predictions of the spread of adolescents’ attitudes and behaviors within classrooms and would open new avenues for studying and developing school interventions. The accuracy of prediction could be enhanced by utilizing a more complete model of the networks of relations channeling peer influence in schools (Veenstra & Laninga-Wijnen, 2022) and allowing researchers to account for possible countervailing effects of different forms of influence. This could be especially beneficial for research based on counterfactual scenarios, which, for example, simulate how attitudes and behaviors spread based on targeting different potentially influential individuals.

The combined impact of friendship influence and normative pressure could have significant implications for school interventions, opening new research avenues. Such interventions could directly use normative pressure to stimulate the adoption of desirable attitudes and behaviors or use the knowledge about normative pressure to spread attitudes and behaviors in so-called network interventions (Valente, 2012). As an example, school interventions could directly increase desirable norms by making sure that students are aware of desirable attitudes and behaviors among peers. Practitioners could collect attitudes among students and present them to students if these results support norms leading to desirable attitudes or behaviors (Perkins et al., 2011). School interventions could exploit a combination of friendship influence and normative pressure to increase resilience toward undesirable norms or to facilitate desirable norms. For instance, if friends are subject to the same normative pressure, their attitudes and behaviors could become reflective of the norm, leaving little room for an adolescent to adopt alternative attitudes or behaviors. However, a friend from a different group who adheres to a different norm could provide an alternative. An intervention could create opportunities for building friendships outside of one’s group, such as using group assignments that would include students from multiple groups (e.g., Boda et al., 2020). Additionally, adolescents could rationally choose the least costly strategy when it comes to forming friendships and adopting attitudes and behaviors. If adolescents are dissatisfied with a norm, they might seek friendships with peers outside the group who do not follow this group norm and support an adolescent in resisting normative pressure. Alternatively, adolescents could find an ingroup friend who also disagrees with a norm and provides support for not following this norm. Interventions supporting such friendships could increase resilience toward harmful normative pressure, or supporting positive norms could increase resilience toward harmful friendship influence.

The knowledge about the role of normative pressure as a form of peer influence could be utilized in network interventions, which use the knowledge about who influences whom to spread desirable attitudes and behaviors (Valente, 2012). An example of such an application is to improve the identification of especially influential adolescents who could spread intervention outcomes among other peers. Thus, the entire success of such interventions crucially depends on the correct identification of influential adolescents. The network approach has proven to be a valuable tool in studying peer influence, particularly in identifying adolescents who occupy central positions within classroom networks and should thus be especially effective in disseminating attitudes and behaviors among their peers (Badham et al., 2019; Zingora et al., 2019). However, if we combine friendship influence and normative pressure, we might identify completely different individuals as being central as compared to an approach relying solely on friendship influence.

**Figure 2.**
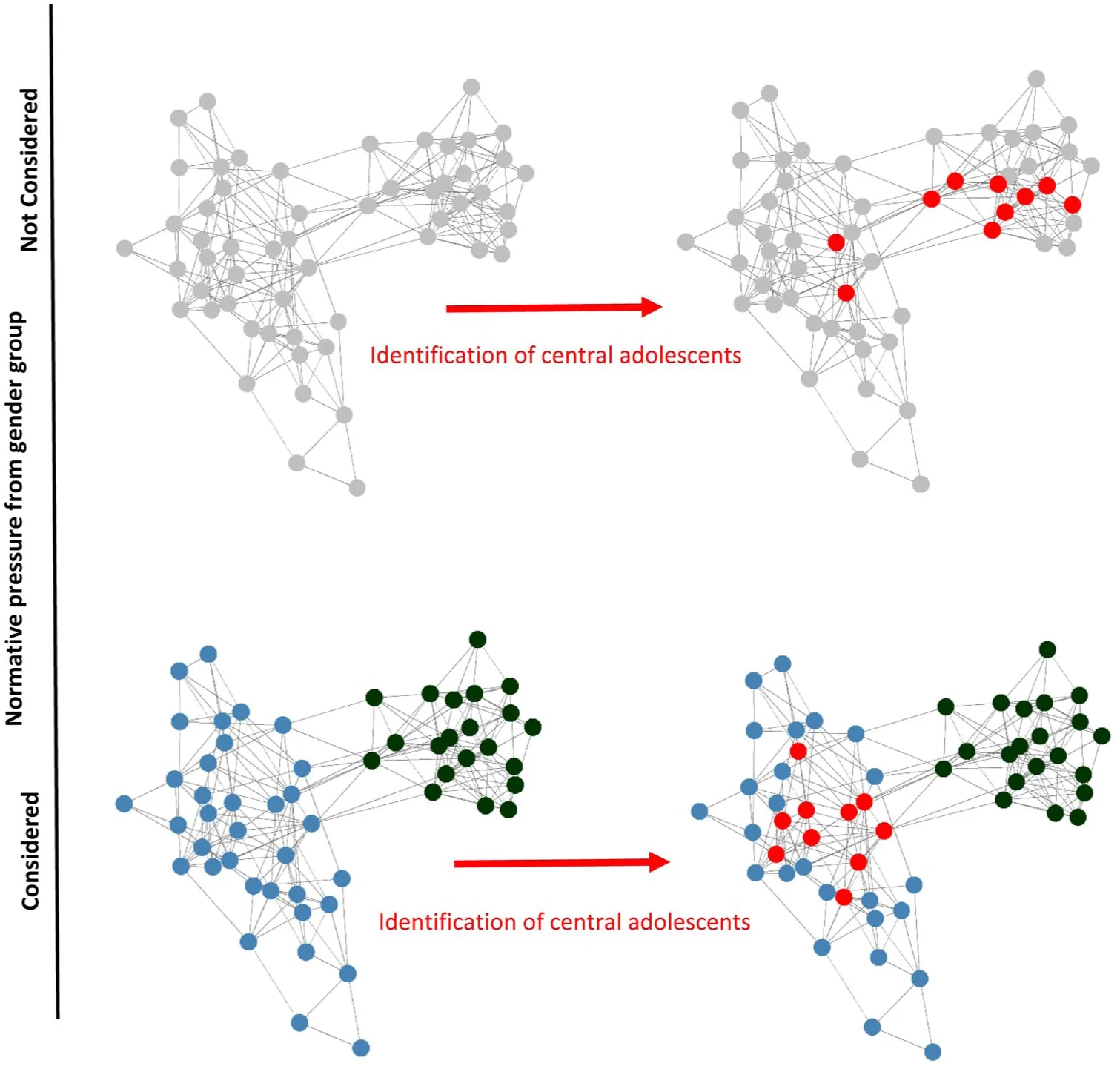
Visualization of the school grade network and highlighting the role of gender in identifying central adolescents. *Note.* The plot contains four network depictions of the same school grade. Circles represent students and links represent friendships. In the top networks, we did not distinguish gender. In the bottom networks, blue dots represent females and dark green males. In the networks on the right, we identified central individuals (having high eigenvector centrality) based on a network of (a) friendship relations (i.e., top network), (b) a combination of friendship relations and belonging to a gender group (i.e., bottom network). Central individuals were labeled with red color.

In Figure 2, we illustrate the importance of considering normative pressure in addition to friendship influence for identifying influential individuals in the same school grade. Following a prominent approach in the literature, we used in this example Eigenvector Centrality of a node as an indicator of the nodes’ influence (Bonacich, 1987, 2007; Borgatti, 2005). Eigenvector centrality captures adolescents whose friends were popular in terms of being nominated as friends by many others. The figure demonstrates how a network construction method that accounts for normative pressure via static group-membership links leads to different conclusions than a method that merely considers friendship nominations. The circles represent adolescents, and the links represent friendships. In the top plots, we constructed a network only using friendship relationships, assuming influence between friends and no influence between non-friends. In the bottom plots, we highlighted adolescents with the same gender using two different colors and assumed that all adolescents with the same gender are automatically connected via links of normative pressure while retaining friendship links as an additional source of peer influence. Links between adolescents of the same gender are not portrayed for the sake of legibility. Thus, in the bottom plots, we assumed that the strongest peer influence was between friends of the same gender, with weaker influence between friends of different genders or between non-friends of the same gender, and no influence between non-friends of different genders. Comparing the plots on the right shows that different individuals would be identified as central in terms of Eigenvector Centrality, depending on whether only friendship influence was investigated (top right plot) or if normative pressure was also considered (bottom right plot). In this particular example, including normative pressure leads to the selection of central individuals only from the numerically dominant group, whereas on the basis of friendship influence alone, a majority of the selected students reside in the smaller group. A more refined approach might balance the number of selected targets in each of the two subgroups more in proportion to the relative size. This example demonstrates how the identification of central individuals, and thus potentially especially influential individuals, can be entirely different if both friendship influence and normative pressure affect adolescents. In the worst case, we might severely overestimate the effects of an intervention if we select adolescents who are supposedly influential, relying on the network of friendship influences alone.

## The Strength of Normative Pressure and Behavioral Frequency

Normative pressure is defined as a tendency to adopt behaviors prevalent in an ingroup. The frequency of a behavior in a particular setting informs adolescents about the behaviors of their ingroup peers and the relevance of that behavior in their school, representing a potentially crucial factor shaping the strength of normative pressure (Cialdini et al., 1991). Thus, an additional goal of our test of normative pressure was to explore how normative pressure depends on the frequency of a behavior within a group. Broadly, this view implies that the higher the frequency of a behavior, the stronger the normative pressure on youth. Conversely, this would mean that normative pressure would have a decreased or no effect on students’ behaviors, if there were no clear norm expressed by a clear majority of ingroup peers adopting a particular attitude or behavior. However, there may also be deviations from majority behavior, for example, if adolescents want to express individuality and uniqueness, possibly supported by similarly deviant friends (Jetten & Hornsey, 2014). Adolescents could even adopt rare behaviors to deviate from a group norm. This would mean that if a behavior is very rare, some adolescents would be inclined to go against the norm and adopt such rare behaviors. Subsequently, such behavior could spread among adolescents via friendship influence, even among those who did not initially seek to deviate in the first place. Our study allows us to test these potentially conflicting implications about the relationship between the frequency of behavior and the strength of normative influence to adopt it.

Alternatively, the frequency of an outcome might not automatically increase normative pressure.^1^ Rather, normative pressure could be increased when peers manifest homogeneous behavior and high consensus (Hogg & Reid, 2006). Deviations from a norm could disrupt the perception of homogeneous normative pressure. Hence, homogeneous behaviors among peers could increase normative pressure, too, regardless of whether behaviors are frequent or not. For example, if all adolescents do their homework occasionally, the behavior is not very frequent but is homogenously endorsed by all adolescents.

A different view could be that the absence of behaviors represents a similarly strong norm as if behavior is very frequent among adolescents. For example, adolescents could be subjected to a similar strength of normative pressure if all peers do their homework very frequently or if all peers never do their homework. We explore all these options to pinpoint the role of the frequency of behavior in shaping the strength of normative pressure on changing students’ behaviors.

## Group Membership and Selection of Attitudes and Behaviors

In addition to investigating the distinction between peer pressure and friendship influence, we explored how the relative strength of each source of influence depends on the nature of the ingroup and the type of attitudes and behaviors. The definition of an ingroup is not as clear-cut as the definition of friends, and the impact of normative pressure across different attitudes and behaviors could be more complex. Generally, adolescents should be influenced by peers who are similar to them in terms of characteristics that define their ingroups (Festinger, 1954; Tajfel, 1982). However, ingroups can be defined based on various characteristics, and adolescents belong to multiple social groups, such as ethnic or gender groups. We investigated the impact of normative pressure on three attitudes and behaviors (academic drive, prejudice, religiosity) and how it varies across two types of ingroups (gender, ethnicity). In this study, we specifically chose gender and ethnicity as group characteristics, because while the gender group is widely studied as an important source of normative pressure in classroom settings across various attitudes and behaviors (Leszczensky & Pink, 2020; Raabe et al., 2019; Schaefer et al., 2013; Stark, 2015), ethnicity is widely used when it comes to ethnicity-related attitudes and behaviors, such as prejudice (Bracegirdle et al., 2022; Stark, 2015; Van Zalk et al., 2013; Zingora et al., 2019). We aimed to select attitudes and behaviors that have been frequently studied using longitudinal social network analysis in a school context and which can be harmful (e.g., prejudice) or beneficial (e.g., academic drive) for the development of adolescents (Bracegirdle et al., 2022; Leszczensky & Pink, 2020; Raabe et al., 2019; Stark, 2015; Van Zalk et al., 2013; Zingora et al., 2019; Zingora, 2024).

## Methods

### Data

We used a dataset called the Friendship and Identity in Schools data (Leszczensky, Pink, et al., 2020). Data comprise responses from 2,838 adolescents (11–14 years old at the first wave; 46% girls; 32% German majority) across 10 schools (29 grades) in Germany. Wave 1 was collected in May 2013, Wave 2 in February 2014, and Wave 3 in November 2014. This dataset is relatively large. Previous research indicates that our sample size yields enough power to test our hypotheses (Stadtfeld et al., 2020). More information about the dataset can be found here: https://fdz.dezim-institut.de/en/metadata_fis.

#### Ethics Information

The data have already been collected (Leszczensky, Pink, et al., 2020); the data collection team was in charge of the ethical aspects and informed consents.

### Measures

#### Gender

Adolescents were asked whether they were girls or boys (0 = boy, 1 = girl).

#### Ethnicity

The ethnic background was assigned based on the birthplace of adolescents, parents, and grandparents. Adolescents were labeled as being of German origin unless there was a migration background. If both parents or grandparents were born abroad, the maternal country of origin took precedence. We used a self-identification measure for adolescents who were born abroad but whose parents and grandparents were not. The list of ethnicities present in the dataset can be found in the Appendix.

#### Friendship Network

Adolescents were asked to list their best friends within their school grade. Friendship networks were directed, which means that we considered who nominated whom as a best friend. We created friendship networks based on these nominations.

#### Group Networks

We created two group networks based on gender and ethnicity. In each group network, adolescents who shared a particular category were labeled as ingroup peers.

#### Dyadic Covariates

We created four dyadic covariates that enabled us to distill the effects of friends from various combinations of gender and ethnicity. Specifically, we created dyadic covariates that specified whether each pair of adolescents belonged to (a) the same gender and ethnicity, (b) different gender and ethnicity, (c) same gender and different ethnicity, (d) different gender and same ethnicity.

#### Academic Drive

Adolescents were asked about the frequency of doing homework on a scale from 1 (never) to 5 (more than 3 hr a day). We assumed that the frequency of homework was related to motivation or drive for academic achievement, which is in line with previous research (Xu, 2005).

#### Prejudice

Adolescents were asked about their attitudes toward different ethnic and religious groups, namely: Germans, Poles, Italians, Turks, Christians, Jews, and Muslims. The scale ranged from 1 (*very positive attitude toward a group*) to 5 (*very negative attitude toward a group*; 6 indicated “*I do not know*”). This allowed us to create a measure of generalized prejudice. Based on ethnicity, religion, and ethnic self-identification, the generalized prejudice measure included only attitudes toward outgroups, not ingroups.

#### Religiosity

Adolescents were asked about the frequency of visiting a place of worship (e.g., church, mosque) on a scale from 1 (*never*) to 5 (*every day*), which was used as a proxy for religiosity (Leszczensky & Pink, 2020).

#### School-Grade Characteristics

We inspected three school-grade characteristics. First, average socio-economic status was estimated using two measures: average amount of pocket money per week and average number of books in a household. Second, students were asked whether they could trust their school-grade peers, which we used to obtain average trust in school-grade peers on a scale from 1 (*completely agree*) to 5 (*completely disagree*). Third, we used information about ethnic diversity in the students’ neighborhoods, represented by the percentage of Germans living in the students’ neighborhoods.

### Analysis Plan

We applied stochastic actor-oriented modeling (SAOM) to the investigation of our hypotheses. Specifically, we used RSiena, which applies SAOM for the analysis of the co-evolution of actor attributes and network relations in longitudinal social network data (Ripley et al., 2024). Generally, SAOM allows studying the simultaneous development of a network structure—represented by friendships in our project—and behavior. The development of network structure is represented by the network evolution function and the development of behaviors or attitudes by the behavioral evolution function. Another important aspect of applying SAOM is allowing us to disentangle friendship influence from friendship selection, that is, forming friendships based on similar attitudes or behaviors in the first place.

SAOM’s central characteristic is modeling the change of the network and the behavior or attitudes from the perspective of agents, in our case, adolescents. SAOMs simulate adolescents’ choices to change their links to peers or adjust their behavior in a series of ministeps (Ripley et al., 2024). In each ministep, one decision is simulated for a randomly selected adolescent, taking into account the characteristics of an adolescent and peers, the network structure, and the network dynamics effects, such as the selection of similar friends. In a ministep, an adolescent can either change a single tie (dissolve, maintain, or create) or change an attitude/behavior by one unit on its respective scale. Decision-making is probabilistic, guided by an evaluation function that reflects the assumptions researchers make about features that define the attractiveness of each option from the agent’s perspective. After each ministep, the network is updated and another adolescent is randomly selected. It is assumed that adolescents have knowledge of the structure of the network, and their decisions are guided by the network dynamics effects. SAOM quantifies how well the simulated changes replicate the observed longitudinal data. Effects constituting the evaluation functions are selected by a researcher and treated as independent variables, the co-evolution of networks and attitudes or behaviors as dependent variables. A combination of simulation and model-fitting techniques is used by RSiena, to estimate the parametrization of the effects that optimizes the fit of the model specified by the researcher to the observed changes of network ties and attitudes in the data.

The estimated effects are represented as log-probability ratios (similar to log-odds). In the results section, we interpreted key significant effects in terms of the odds of changing an attitude or behavior by one unit on the attitude scale in the direction of the parameter sign, rather than staying stable. Following the RSIENA manual, these odds can be computed using the formula: e^β/r^ (Ripley et al., 2024, p. 207), where β is the parameter estimate and *r+*1 the number of scale points on the attitude scale. Other than in common logistic models, the number of scale points factors here into the computation of the effect size, because the parameter estimate is derived from the magnitude of the overall observed change of an attitude in between two waves, which implies a larger effect if an attitude changed by a certain number of scale points on a scale with few points compared to a scale with many points.

We estimated a series of models to disentangle different types of peer influence (see Figure 1). In *Model 1*, we inspected friendship influence by inspecting the effect of average attitudes and behaviors among friends instead of normative pressure. In *Model 2*, we included the effect of normative pressure, captured by the tendency to change attitudes and behaviors in the direction of average attitudes and behaviors among peers from the same group. To this end, we used two static group networks: gender and ethnic networks. Normative pressure was found if adolescents were influenced in the direction of attitudes and behaviors prevalent among ingroup peers. In *Model 3*, we combined Models 1 and 2. Significant effects of both normative pressure and friendship influence in Model 3 would mean that both have a unique impact on the evolution of attitudes and behaviors. If only normative pressure or friendship influence was significant in Model 3, but both effects were significant in Models 1 and 2, it would mean that they could be mistaken for each other due to their overlap, but the significant effect in Model 3 would be superior to the non-significant effect. Each series of models was estimated for each outcome. Moreover, we studied normative pressure based on gender and ethnic group in separate models, thus, each series of models was estimated twice. Gender and ethnic group membership were investigated in separate models to avoid convergence issues but to increase the robustness of our findings. This means that each series of models was estimated six times, resulting in 30 models.

Our next aim was to investigate the influence of ingroup friends. To this end, we used four dyadic covariates, which captured whether friends belonged to the same or a different gender or ethnic group. We weighted the social influence of friends by these dyadic covariates, which allowed us to distill the unique social influence of friends belonging to all these groups. *Model 4* contained effects that captured the influence of ingroup and outgroup friends. *Model 5* combined the influences of ingroup and outgroup friends and normative pressure effects. If we found that friendship influence depended on the ingroup/outgroup membership of friends in Model 4 but not in Model 5, it would mean that testing only for the influence of ingroup friends could result in an inaccurate conclusion that ingroup friends, not ingroup peers, are particularly influential. If we found support for ingroup friends being particularly influential in Model 5 as well as normative pressure, it would mean that ingroup friends and normative pressure yielded unique effects on the development of adolescents.

We estimated a range of covariate effects that may influence the development of outcomes or friendship networks. These variables can serve both as predictors of interest and as controls for potential confounding. The full models can be found in the Appendix. Here, we provide guidance on the interpretation of effects driving the development of behaviors, that is, the behavioral evolution function. First, we included linear and quadratic shape effects, representing the general development of an outcome in a school grade. Second, we estimated the effect of average behavior in the whole school grade, which captured the overall influence of all peers as well as regression to the mean. Third, in models focused on gender, we estimated the effect of gender. This effect represented systematic differences in the development of behaviors between girls and boys. For example, a significant gender effect would mean that girls, on average, changed their academic drive differently than boys. In the network evolution function, we also estimated whether students created friendships with peers of the same gender, ethnicity, or attitudes and behaviors. We did not estimate the role of ethnicity in a behavioral function because ethnicity contained multiple categories, and including separate dummy variables to control for each ethnicity effect could have hampered the convergence of the model. The effects crucial for testing our hypotheses should be interpreted in light of these controls. This is illustrated in the following example of Model 3, which focuses on academic drive and gender. SAOM estimates students’ decision-making on whether and how to change their academic drive. Based on the effects described above, SAOM would estimate whether a student changed academic drive to become more similar to same-gender peers or friends or whether an observed change in academic drive was due to the fact that all peers changed in the same direction (linear and quadratic shape effects), that students tended to resemble academic drive of all peers, not necessarily of same-gender peer or friends (average behavior effect), or that girls changed academic drive in one way and boys in another (gender effect). At the same time, we controlled for various effects that reflect changes in a network, such as a tendency to build friendships with similar peers in terms of gender, ethnicity, attitudes, or behaviors.

To address the issue of missing data, we conducted multiple imputations using the mice package implemented in R (van Buuren & Groothuis-Oudshoorn, 2011). We used predictive mean matching to impute the data, except for the friendship networks. The imputations depended on a variety of relevant information. The information used in the imputations depended on the outcome; for each outcome, we used outcome value at all three time points, indegree (incoming friendship nominations) at all three time points, average outcome value in the whole school grade at all three time points, average outcome value in the ethnic and gender groups at all three time points, gender, and ethnicity. We imputed all information except friendship networks, which would require a different approach (Krause et al., 2018). For each school grade, we imputed the dataset five times and combined the results using Rubin’s rules. The implementation of Rubin’s rules was done outside of the mice package, and we adopted the code of Krause and Iashina (2019). The effects were included in the model only if the standard error of each effect was not higher than 20. If it was, we inspected all results and used results based on a particular imputation only if the standard error was below 25 and it did not concern effects that tested our hypotheses (i.e., normative pressure or social influence effects). If a model did not converge, we inspected the reasons. If the reason was the effect of an average outcome in the whole school grade, which could be collinear with the linear shape effect, we dropped this effect. If the model did not converge for other reasons, it was not included in the analysis. The effects could differ across different school grades, thus, we estimated a model for each school grade separately and combined these results within a meta-analysis (Snijders & Baerveldt, 2003) using the metafor package implemented in R programming (Viechtbauer, 2010). More information about the proportion of missing values can be found in the Appendix.

To test hypotheses related to the varying effects of normative pressure on the development of behaviors, we applied meta-regression and the Knapp and Hartung (2003). First, using the linear effect, we tested whether the higher frequency of behaviors was related to stronger normative pressure. Second, we added the quadratic effect, which tested whether normative pressure is stronger when behavior is, on average, very frequent or infrequent. Third, we added the effect of variance of behavior in a school grade, which tested whether normative pressure has a stronger effect on the development of behaviors when there is a predominant behavior in a school grade, and variance is hence low. We followed this procedure using Model 3, a parsimonious model that contained both friendship influence and normative pressure. These hypotheses might be more relevant for behaviors than attitudinal outcomes, such as prejudice. The reason is that no value of attitudes could be labeled as the absence of an attitude in a school grade. For example, if all students report no prejudice, that would not indicate the absence of this attitude. Nevertheless, we repeated the test for prejudice for the sake of completeness.

We also investigated whether important differences between school grades affect the effect of normative pressure on behaviors. We investigated whether average socio-economic status, average trust toward peers in a school grade, and average neighborhood diversity predicted the strength of normative pressure. Again, we applied meta-regression in this task and focused only on Model 3.

This research was preregistered and its record can be found here: https://osf.io/xmszj/?view_only=b3e1a7ea0b6d44b78a6ad95532630120. The analysis code developed in this project can be found here: https://osf.io/xz4kf/?view_only=8918fed9278e476a863f6616df20c45d. More detailed results, including tests of the model fit, mathematical representation of the effects, complete SAOM results, deviations from the preregistration, and descriptive results can be found in the Appendix.

## Results

**Figure 3.**
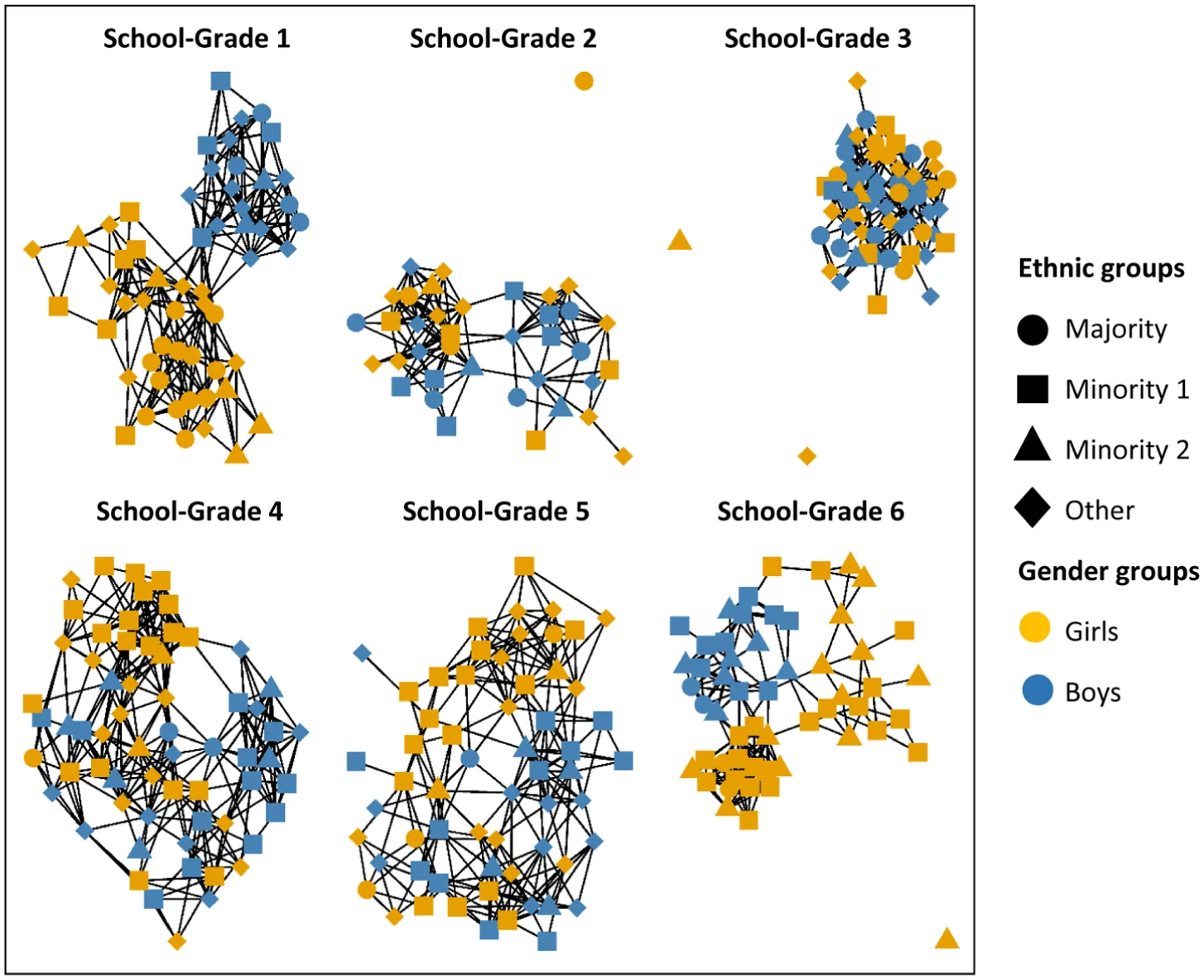
Six examples of the school-grade networks highlighting selection tendencies.

We analyzed 29 German school grades, containing 2,838 adolescents. Examples of social networks of adolescents’ friendships are portrayed in Figure 3. In this figure, we illustrated six school grades at the first time point of our investigation. Adolescents were portrayed using colored shapes; different shapes reflect different ethnicities and different colors gender. Adolescents were linked together via friendships illustrated with black lines. The figure visualizes homophily tendencies among students, as students of the same gender (i.e., same color) are grouped together. Gender homophily in friendship choices was also confirmed by our SAOM results (for the full results, see Appendix).

**Figure 4.**
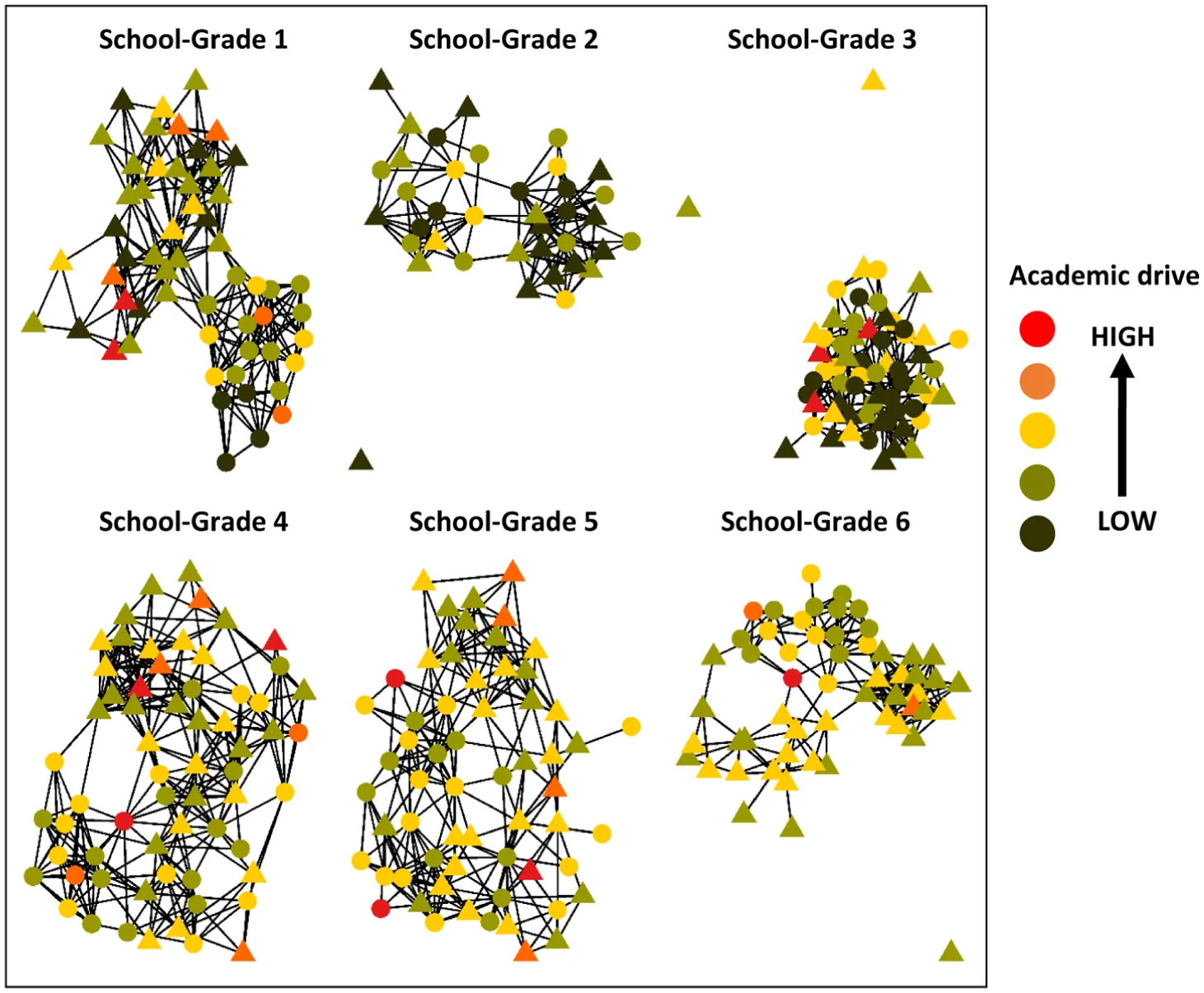
Six examples of the school-grade networks highlighting academic drive among students.

Figure 4 portrays the same set of school grades as Figure 3. In Figure 4, we highlighted the values of one of the outcomes (i.e., academic drive) among students. Students tend to have a similar academic drive as peers around them. The question is why are these students similar and do they become even more similar in time because of the influence? Or do students rather bond with peers who have a similar academic drive in the first place? Even if they influence each other, are students influenced only by friends or also by other peers from the same social groups? We aimed to answer these questions by analyzing the network data, characteristics, attitudes, and behaviors of adolescents using Stochastic Actor-Oriented Modeling.

Table 1 shows correlations between variables. The correlations between outcomes (i.e., academic drive, prejudice, religiosity) at different time points were approximately 0.5. This suggests that meaningful changes occurred over time, providing the necessary variation for modeling the dynamics of these outcomes.

**Table 1.** Means, Standard Deviations, and Correlations.

Variable	*M*	*SD*	1	2	3	4	5	6	7	8	9	10	11	12	13	14	15	16
1. Gender	1.54	0.50																
2. N same-gender peers	42.21	19.49	.15**															
3. N same-ethnicity peers	18.56	18.69	−.00	.51**														
4. N friends T1	4.22	3.88	−.01	.34**	.21**													
5. N same-gender friends T1	3.55	3.35	.00	.35**	.22**	.95**												
6. N different-gender friends T1	0.52	1.11	−.03	.12**	.06**	.49**	.21**											
7. N same-ethnicity friends T1	1.26	1.93	−.01	.27**	.56**	.58**	.58**	.23**										
8. N different-ethnicity friends T1	2.80	3.05	−.00	.26**	−.09**	.85**	.81**	.45**	.09**									
9. Academic drive T1	2.41	0.89	−.07**	.08**	.03	.03	.05*	−.01	.04	.02								
10. Academic drive T2	2.37	0.89	−.09**	.05*	.03	.03	.04	.00	.06**	−.00	.46**							
11. Academic drive T3	2.39	0.89	−.15**	.03	.01	.03	.04	−.02	.05*	.00	.38**	.50**						
12. Prejudice T1	3.54	1.00	−.08**	.04	−.05*	.06*	.07**	.01	−.09**	.14**	.02	.03	.03					
13. Prejudice T2	3.70	0.94	−.08**	.03	−.03	−.03	−.03	−.01	−.07**	.02	−.00	.00	−.00	.46**				
14. Prejudice T3	3.68	0.94	−.13**	.04	.01	−.05	−.04	−.03	−.05*	−.02	.02	.03	.00	.49**	.55**			
15. Religiosity T1	2.59	1.21	−.00	.09**	.05	.11**	.16**	−.06*	.17**	−.01	.11**	.12**	.10**	.06*	.05	.08*		
16. Religiosity T2	2.59	1.20	−.01	.10**	.10**	.08**	.10**	−.02	.14**	.00	.08**	.13**	.10**	.01	.01	.00	.69**	
17. Religiosity T3	2.56	1.21	.02	.08**	.11**	.07**	.09**	−.02	.14**	−.00	.09**	.12**	.14**	−.00	.00	.05*	.59**	.68**

*Note. M* and *SD* are used to represent mean and standard deviation, respectively.

* indicates *p* < .05. ** indicates *p* < .01.

*M* and *SD* denote mean and standard deviation, respectively.

T1 represents Wave 1 and T2 represents Wave 2.

### Friendship Influence and Normative Pressure

**Figure 5.**
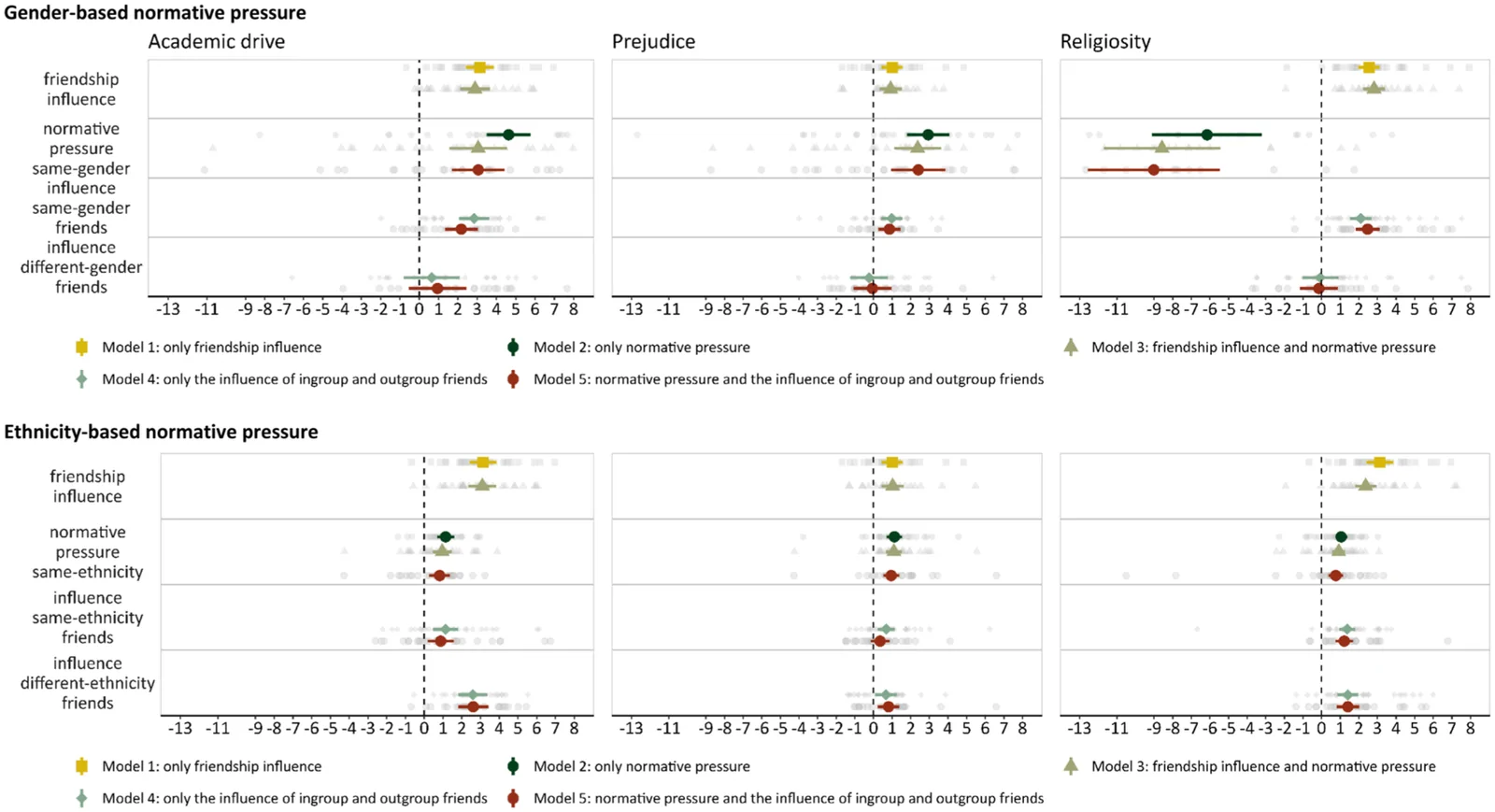
The pooled effects (with 95% confidence intervals) represent the log-odds chance of changing an outcome to become more similar to friends (friendship influence), group members (normative pressure), ingroup friends (influence of ingroup friends), outgroup friends (influence of outgroup friends). *Note.* Colors and shapes represent different models. The effects presented with the same color and same shape thus represent estimates from the same model.

The results supported our hypotheses that peer influence consisted of both friendship influence and normative pressure. Figure 5 describes the sizes of the key effects—friendship influence, normative pressure, and friendship influence separated into the influence of ingroup and outgroup friends. The models are presented separately for each outcome (academic drive, prejudice, religiosity) and a type of group membership (gender, ethnicity). The models were built from less complicated to the most exhaustive Model 5, mapping the consequences of studying friendship influence and normative pressure together. To estimate the effects, we first estimated each school grade separately and then combined the results within a meta-analysis. Thus, each effect presented in Figure 5 in color represents an estimate based on the meta-analysis together with 95% confidence intervals of an estimate. Estimated effects for each school grade are presented in grey. Figure 5 illustrates the changes in effect sizes across models; full results and the heterogeneity tests of key effects can be found in the Appendix.

Both friendship influence and normative pressure were significant predictors of academic drive, prejudice, and religiosity. The effects were robust since they were significant regardless of whether we estimated friendship influence and normative pressure separately (Models 1 and 2) or together in one model (Model 3; see Figure 5; for the sake of eligibility, we present only estimates from Model 3 in this paragraph).

Specifically, friendship influence and normative pressure predicted outcomes in all tested models and regardless of whether group membership was based on gender (*academic drive friendship influence* estimate = 2.89, *SE* = 0.40, *p* < .001; *academic drive normative pressure* estimate = 2.89, *SE* = 0.40, *p* < .001; *prejudice friendship influence* estimate = 0.92, *SE* = 0.31, *p* < .001; *prejudice normative pressure* estimate = 2.38, *SE* = 0.64, *p* < .001; *religiosity friendship influence* estimate = 2.82, *SE* = 0.30, *p* < .001; *religiosity normative pressure* estimate = −8.57, *SE* = 1.60, *p* < .001) or ethnicity (*academic drive friendship influence* estimate = 3.10, *SE* = 0.37, *p* < .001; *academic drive normative pressure* estimate = 0.96, *SE* = 0.26, *p* < .001; *prejudice friendship influence* estimate = 1.03, *SE* = 0.30, *p* < .001; *prejudice normative pressure* estimate = 1.10, *SE* = 0.21, *p* < .001; *religiosity friendship influence* estimate = 2.37, *SE* = 0.29, *p* < .001; *religiosity normative pressure* estimate = 0.92, *SE* = 0.17, *p* < .001). This means that adolescents’ outcomes changed in the direction of an average outcome among friends as well as among peers from the same social group. This is true for all of our tests except one. In the case of religiosity, we found a negative impact of normative pressure. Thus, adolescents’ religiosity tended to deviate from the average religiosity among peers of the same gender.

These effects can also be understood in terms of the probability of an increase in behaviors or attitudes compared to staying constant in the event of a mini-step (Ripley et al., 2024). The estimated effect of friendship influence on academic drive is about 3 for both gender and ethnicity, 1 for prejudice, and 2.5 for religiosity. Academic drive, prejudice, and religiosity ranged from 1 to 5. This would mean that if all friends have higher academic drive, prejudice, or religiosity, a student is 2.06 times more likely to increase academic drive by 1 unit, 1.28 more likely to increase prejudice by 1 unit, and 1.87 more likely to increase religiosity by 1 unit than staying stable. In the case of normative pressure, the effects varied depending on whether groups were based on gender or ethnicity. In the case of gender groups, if all same-gender peers have higher academic drive, prejudice, or religiosity, a student is 2.06 times more likely to increase academic drive by 1 unit, 1.81 more likely to increase prejudice by 1 unit, and more likely to decrease religiosity by 1 unit than staying stable. In the case of gender groups, if all same-ethnic peers have higher academic drive, prejudice, or religiosity, a student is 1.27 times more likely to increase academic drive by 1 unit, 1.32 more likely to increase prejudice by 1 unit, and 1.26 times more likely to increase religiosity by 1 unit than staying stable. These effects highlight preferences when SAOM simulates a student's decision-making. Over the course of an analysis that simulates many mini-steps, these effects can reflect significant changes in behaviors and attitudes among all students.

In a robustness check, we tested whether an alternative calculation of normative pressure that could be more appropriate for extremely skewed attitudes or behaviors would produce negative normative pressure of the same-gender group in the case of religiosity. This calculation would capture whether exposure to religious behavior—regardless of whether it is prevalent among same-gender peers—would affect the adoption of the religious behavior. This analysis produced again the same result (*religiosity friendship influence estimate based on average alter* = 0.17, *SE* = 0.06, *p* = .003; *religiosity normative pressure estimate based on average alter* = −1.02, *SE* = 0.51, *p* = .044).

### The Influence of Ingroup and Outgroup Friends

When we distinguished whether friendship influence was yielded by ingroup or outgroup friends, we found that both normative pressure and some form of friendship influence—either the influence of ingroup, outgroup friends, or both—affected the development of adolescents’ outcomes. We found that friends from the same group influenced adolescents’ outcomes in all scenarios (Model 5 in Figure 5) in the case of gender-ingroup friends (academic drive estimate = 2.18, *SE* = 0.43, *p* < .001; prejudice estimate = 0.86, *SE* = 0.34, *p* = .005; religiosity estimate = 2.46, *SE* = 0.33, *p* < .001) and in two out of three scenarios in the case of ethnic-ingroup friends (academic drive estimate = 0.88, *SE* = 0.36, *p* = .013; prejudice estimate = 0.36, *SE* = 0.26, *p* = .174; religiosity estimate = 1.22, *SE* = 0.25, *p* < .001). Inspecting the non-significant influence of ethnic-ingroup friends on prejudice, we found that they were seemingly influential in Model 4 (estimate = 0.69, *SE* = 0.23, *p* = .003), thus, until we accounted for normative pressure based on ethnicity. Thus, in the case of prejudice, ethnic-ingroup friends influenced prejudice but only to the same extent as other ethnic-ingroup peers.

Gender-outgroup friends did not influence adolescents’ outcomes (academic drive estimate = 0.95, *SE* = 0.76, *p* *=* .212; prejudice estimate = −0.06, *SE* = 0.52, *p* = .915; religiosity estimate = −.16, *SE* = 0.53, *p* *=* .758), contrasting to ethnic-outgroup friends who influenced all outcomes (academic drive estimate = 2.61, *SE* = 0.41, *p* < .001; prejudice estimate = 0.81, *SE* = 0.30, *p* = .006; religiosity estimate = 1.41, *SE* = 0.31, *p* < .001 ).

In the case of academic drive, students were 1.72 times more likely to increase academic drive by 1 unit than staying stable if all same-gender friends scored higher, 1.25 times more likely if all same-ethnic friends scored higher than a student, and 1.92 times more likely if all different-ethnic friends scored higher than a student. In the case of prejudice, prejudice was 1.24 times more likely to increase by 1 unit than staying stable if all same-gender friends scored higher and 1.22 times more likely if all different-ethnic friends scored higher than a student. In the case of religiosity, religiosity was 1.85 times more likely to increase by 1 unit than staying stable if all same-gender friends scored higher, 1.36 times more likely if all same-ethnic friends scored higher than a student, and 1.42 times more likely if all different-ethnic friends scored higher than a student.

### The Strength of Normative Pressure and Behavioral Frequency

**Figure 6.**
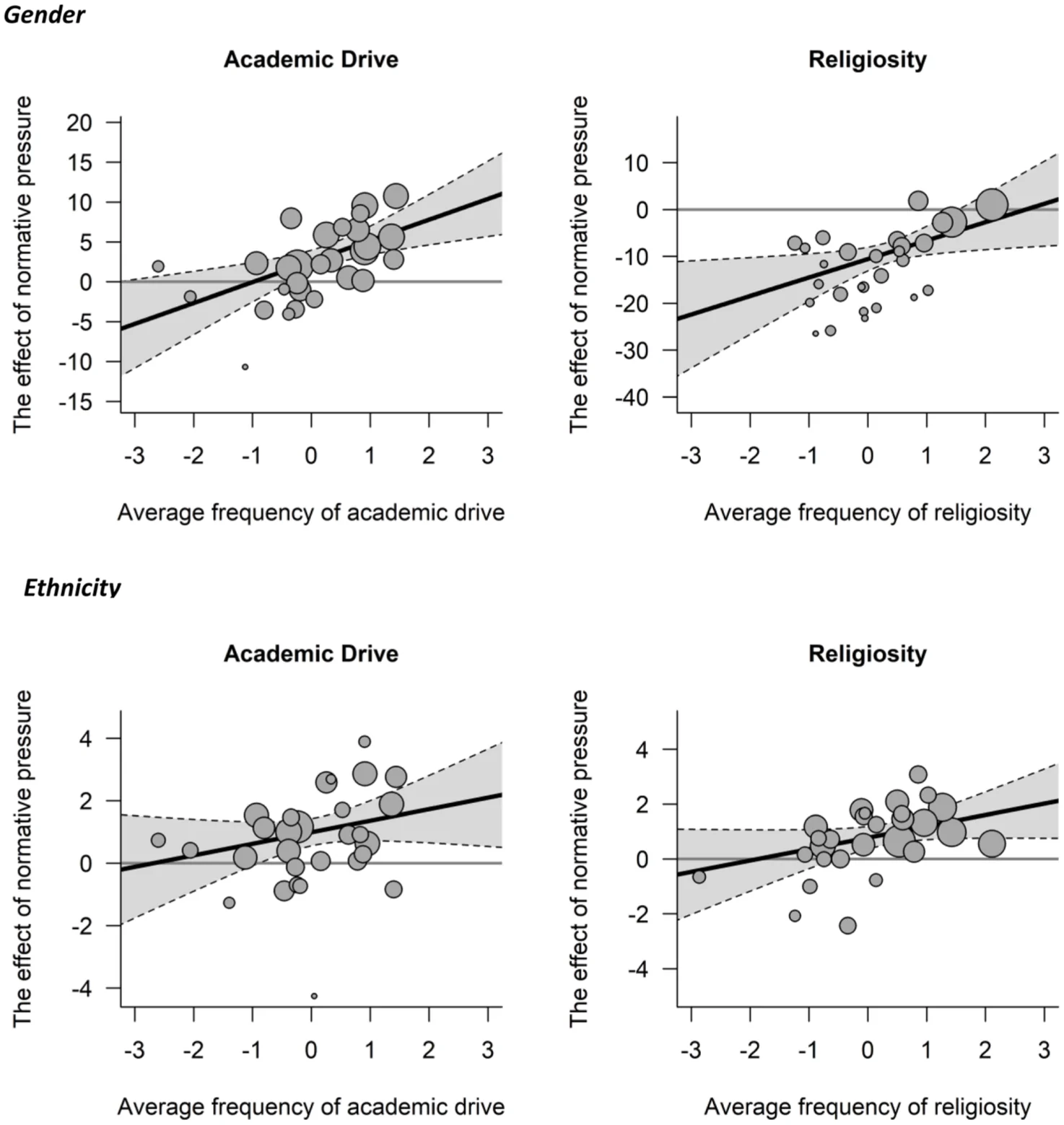
Interaction between the average frequency of an outcome (*y*-axis) and the strength of normative pressure (*x*-axis) based on gender (top plots) and ethnicity (bottom plots). *Note.* Plots were prepared using *metafor* package implemented in R. The bubble size represents the weight of a particular estimate; the weight was determined by the standard error associated with an estimate. In the case of religiosity and normative pressure based on ethnicity (bottom right plot), three observations were dropped to increase the legibility of the figure; the regression line was based on all observations. Average frequencies of academic drive and religiosity were abstracted for each school grade and then scaled and centered. These average frequencies are portrayed on the X-axis. We restricted the range of the X-axis; this might reflect different average frequencies due to scaling. Before scaling, the average frequency of academic drive ranged from 1.56 to 2.82, and the average frequency of religiosity ranged from 1.60 to 3.22, both on a scale from 1 to 5.

Normative pressure was linked to the development of adolescents’ attitudes and behaviors. In this part, we tested the role of the frequency of a particular behavior on the effect size of normative pressure on the development of academic drive, prejudice, and religiosity. As shown in Figure 6, normative pressure increased as a function of the frequency of a particular outcome in a school grade. Especially in the case of gender, the frequency of an outcome increased the coefficient for normative pressure with respect to academic drive (*b* = 2.61, *SE* = 0.80, *p* = .003) and religiosity (*b* = 3.94, *SE* = 1.62, *p* *=* .023), not prejudice (*b* = −0.12, *SE* = 1.20, *p* *=* .921). In the case of ethnicity, the predictive role of frequency was less pronounced (academic drive, *b* = 0.37, *SE* = 0.25, *p* *=* .151; religiosity, *b* = 0.41, *SE* = 0.22, *p* *=* .070; prejudice, *b* = 0.60, *SE* = 0.38, *p* *=* .131). The quadratic effect of the frequency of an outcome in a school-grade was not significant (gender: academic drive, *b* = 0.69, *SE* = 0.63, *p* *=* .285; religiosity, *b* = 1.43, *SE* = 0.98, *p* *=* .156; prejudice, *b* = −0.32, *SE* = 0.85, *p* *=* .709; ethnicity: academic drive, *b* = −0.08, *SE* = 0.20, *p* *=* .699; religiosity, *b* = −0.17, *SE* = 0.15, *p* *=* .263; prejudice, *b* = −0.14, *SE* = 0.23, *p* *=* .557). The variance of an outcome was not a significant indicator of normative pressure effect either (gender: academic drive, *b* = −0.72, *SE* = 0.81, *p* *=* .379; religiosity, *b* = −1.36, *SE* = 1.34, *p* *=* .323; prejudice, *b* = 0.21, *SE* = 1.01, *p* *=* .841; ethnicity: academic drive, *b* = −0.17, *SE* = 0.21, *p* *=* .438; religiosity, *b* = 0.14, *SE* = 0.22, *p* *=* .551; prejudice, *b* = 0.16, *SE* = 0.35, *p* *=* .649). Taking these complex results together, normative pressure yielded by gender groups increases as a function of increasing frequency of behavior measures—academic drive and religiosity—nor attitudinal measures, specifically prejudice.

### Effect Sizes and Potentially Important Characteristics of School-Grades

We inspected four potentially important differences between school grades that could be linked to various effect sizes of normative pressure and friendship influence. Specifically, we inspected proxies of average socio-economic status, neighborhood diversity, and trust in school-grade peers. As shown in Table 2, none of these differences was linked to the effect size of normative pressure and friendship influence.

**Table 2.** The Effects of Potentially Important School-Grade Characteristics on Normative Pressure and Friendship Influence in a School-Grade.

The average attribute in a school grade	Academic drive				Prejudice				Religiosity			
	Gender		Ethnicity		Gender		Ethnicity		Gender		Ethnicity	
	NP	FI	NP	FI	NP	FI	NP	FI	NP	FI	NP	FI
Pocket money	−1.29	−0.12	−0.40	−0.14	1.01	−0.79	0.35	−0.75	−0.84	−0.17	−0.24	−0.15
Number of books	−0.84	0.71	−0.34	0.60	−0.59	0.20	−0.20	0.20	−0.08	−0.03	−0.24	−0.15
Trust in school-grade peers	0.24	−0.23	−0.26	−0.18	0.59	−0.03	0.20	0.01	−3.42	0.32	0.24	0.20
Neighborhood diversity	0.78	−0.58	0.13	−0.46	1.10	−0.54	−0.50	0.38	0.38	0.34	0.20	0.33

*Note.* NP = normative pressure; FI = friendship influence.

Pocket money and the number of books were used as proxies for socio-economic status. The estimates represent meta-regression coefficients when the average attribute in a school-grade (shown in the left column) was a predictor of normative pressure and friendship influence estimates for each school grade.

All effects were non-significant (*p* > .05).

## Discussion

Research on peer influence that capitalized on social network science largely assumes that adolescents influence each other if they form an interpersonal relationship, especially friendship, and thus that attitudes and behaviors propagate among adolescents along friendship lines (Raabe et al., 2019; Steglich et al., 2006; Zingora et al., 2019). Our research suggests that peer influence extends beyond adolescents' closest interpersonal relationships. Adolescents were substantially influenced not only by friends but also by peers who were not their friends but who belonged to the same social groups as the adolescents. Thus, peer influence encompassed both friendship influence and normative pressure, underscoring its multifaceted and nuanced nature. The understanding of who influences whom in schools, who wields especially large influence, how various critical outcomes propagate through adolescent networks, or, in other words, the overall picture of peer influence, is more accurate if we consider both normative pressure and friendship influence. In this research, we showed that both friendship influence and normative pressure complemented each other in shaping the development of adolescents’ academic drive, prejudice, and religiosity.

Furthermore, our results indicate that friends exerted influence over adolescents only if they belonged to the same gender group and regardless of friends’ ethnicity. This pattern has been found in all combinations of outcomes and group memberships (i.e., gender and ethnicity), except in the case of prejudice and ethnic group membership. The influence of ethnic ingroup friends on prejudice was explained by the normative pressure exerted by all peers from the same ethnic group. Thus, ethnic ingroup friends were influential but only to the same extent as other peers from the same ethnic group. Nevertheless, we replicated prior findings that ingroup friends typically influence adolescents (Bracegirdle et al., 2022; Raabe et al., 2019). However, our findings indicated that not all social categories that are commonly used to distinguish ingroup and outgroup friends matter for their friendship influence.

Taken together, this implies that students were influenced by same-gender and not by different-gender friends. Thus, boys would not be influenced by girls and vice versa. In the case of ethnicity, we again found that same-ethnicity friends did influence students, except for prejudice. However, different-ethnicity friends also influenced students across all outcomes. It is possible that these students remained influential because they often shared gender with students, which granted them influence. This calls for more interdisciplinary future research that should pinpoint which social categories matter for labeling peers as ingroup and outgroup, especially since these categories naturally overlap. One strategy that has been used is to focus on the outcome in question. For example, in the case of ethnic prejudice, the social categorization is typically based on ethnicity (Bracegirdle et al., 2022). This assumption seems problematic when members of a social group, such as a school, engage in ongoing interactions and social influence is expected to jump from person to person, depending on the topic. In this vein, we showed that friendship influence and normative pressure were found across a range of topics.

We took one step further in understanding normative pressure among youth and investigated how its effects depended on the frequency of outcomes among all students. Particularly in the case of gender groups, we corroborated the theory on norms (Cialdini et al., 1991)—the higher the frequency of an outcome, the stronger the normative pressure. Moreover, at the very low levels of an outcome in a school grade, normative pressure had a negative effect on outcomes, which means that students tended to deviate from the norm. Several mechanisms could explain this tendency, such as the motivation to be unique (Jetten & Hornsey, 2014). Importantly, this finding points toward the occurrence of non-existent outcomes among adolescents and the subsequent spread of these outcomes. Some adolescents might adopt certain behaviors only to deviate from the norm, functioning as a seed of new forms of behavior in a school grade. Adoption of such behaviors could rupture norms and open the door for further spread along friendship lines. Thus, our research suggests that at the very low levels of an outcome, adolescents tend to be affected by norms but in a negative direction. When an outcome becomes more frequently adopted, it can spread along friendship lines. Even then, adolescents are still influenced by friends and increasingly by normative pressure. Our research not only revealed that normative pressure affects adolescents alongside friendship influence, and that normative pressure increases with a higher frequency of an outcome among adolescents, but also opens the door for future research on how some outcomes appear among adolescents and subsequently spread. This understanding could help us devise strategies to effectively intervene among adolescents using processes that are responsible for the adoption of behaviors.

Our findings consistently showed the importance of normative pressure on the development of adolescents’ attitudes and behaviors. We found, however, one exception. Adolescents’ religiosity tended to deviate from the religiosity of same-gender peers. The negative effect of normative pressure on religiosity disappeared with an increasing frequency of religious behaviors among peers. The reason might be that the adoption of rare behavior signifies a tendency to differentiate from the rest of the peers (Cialdini et al., 1991). Such a tendency would lose its meaning when behaviors become more common. Alternatively, the negative effect of normative pressure on religiosity might reflect a situation in which minority subgroups were influenced in the direction of adopting religiosity and larger subgroups were not. For example, previous research showed that religion is more important for Muslim minority adolescents when they form a sufficiently large minority in a school (Leszczensky, Flache, et al., 2020). Thus, changes in religiosity of such minority adolescents could be affected by their religious subgroup, while it would seem that they deviate from the rest of their gender group. We are looking forward to more research on how religiosity spreads among adolescents and how rare behaviors emerge in schools, while we encourage researchers to consider both friendship influence and normative pressure in shaping adolescents’ religiosity.

The network science approach has reshaped our understanding of how adolescents’ interpersonal relationships evolve and influence each other. One major strength of our paper is an advanced analytical approach that accounted for crucial tendencies that could obscure our findings, such as separating social influence from a tendency to select similar friends or dynamic processes behind changes in friendships (Snijders et al., 2010; Steglich et al., 2010). While network science traditionally assumed that outcomes spread via interpersonal relationships, particularly friendships (Boda, 2018; Schaefer et al., 2013; Steglich et al., 2006), we showed a flow of influence among those adolescents who belonged to the same social group. These findings have significant implications for studying peer influence. For instance, as we illustrated in Figure 2, centrality indices designed to identify influential adolescents would target entirely different individuals when accounting for both normative pressure and friendship influence. We recommend that researchers investigate both friendship influence and normative pressure simultaneously and carefully consider the consequences of studying only one type of peer influence.

### Implications and Future Directions

Our findings have significant implications for school interventions, which should consider both friendship influence and normative pressure if they aim to spread attitudes or behaviors among students. For example, an intervention might successfully induce desirable norms. However, after an adolescent is back in a classroom, friendship influence can resume its effect, causing an adolescent to change his attitude or behavior back to its original state. Intervention could use information about how friendship influence and normative pressure shape attitudes and behaviors, and consider their mutual impact on the effectiveness of an intervention. For example, targeting students who are well-positioned to spread attitudes—due to their friendships and group memberships—can increase the effectiveness of an intervention. Several intervention programs attempted to select influential individuals to spread attitudes and behaviors in schools (Campbell et al., 2008; Paluck et al., 2016). We argue that such selection would be more accurate if normative pressure is considered along with friendship influence. Moreover, we suspect that the prediction of intervention consequences cannot be accurate if we do not consider important forms of peer influence.

Interventions that do not distinguish between friendship influence and normative pressure could also have unintended and undesirable consequences, including polarization or segregation. Interventions that focus on friendship influence alone might fail to change the norm of one group (e.g., girls) while being successful in changing a norm in a second group (e.g., boys), for example, because they underestimate the enforcement of an undesirable peer norm in one of the two groups. Such a difference in norms post-intervention could start polarization, whereby group members would be reinforcing each other’s attitudes or behaviors, resulting in two polarized groups. At the same time, a widening gap between the preferences of the two groups could result in a dissolution of intergroup friendships, resulting in segregation. Considering both friendship influence and normative pressure could thus be critical for school interventions.

Another contribution of our paper is that we studied three important yet distinct attitudes and behaviors—academic drive, prejudice, and religiosity—combined with social categorization based on two broadly studied students’ characteristics—gender and ethnicity. In this way, we provide robust and complex findings, increasing confidence in our conclusions.

This advancement in understanding peer influence opens new avenues for future research. We encourage future research to examine more closely how attitudes and behaviors occur in a school for the first time and subsequently spread. Some behaviors might be adopted to deviate from peers in the first place, which implies negative normative pressure, and subsequently spread via friendships up to a point when a behavior becomes normative. Therefore, we recommend paying attention to the time-related development of attitudes and behaviors and consider both normative pressure and friendship influence.

Furthermore, we found general principles of who influences whom in schools. However, there might be key individuals who are more influential than others, steering the development of many peers (Zingora et al., 2019). Such individuals could represent injunctive norms of the group, being more visible than other members of the same group, or be just strategically positioned in the network to influence others (e.g., Cialdini et al., 1991; Dijkstra et al., 2008; Laninga-Wijnen et al., 2019). In our view, one of the tasks of future research should be the identification of such especially influential individuals. Norms have long been considered to be of importance in this endeavor (Badham et al., 2019; Paluck & Shepherd, 2012; Paluck et al., 2016; Zingora et al., 2019). Now, researchers might study this question while capitalizing on the benefits of the longitudinal social network approach that captures crucial dynamic tendencies affecting normative pressure, such as friendship influence.

Moreover, as outlined above, we believe that one of the tasks that remains is to develop a more nuanced understanding of the importance of different social categories for social influence. Researchers must select some categories that they expect to grant social influence to peers in a given study, but the list of potential categories that could be theoretically important is extensive. In this effort, researchers are usually guided by the topic of investigation (Bracegirdle et al., 2022; Leszczensky & Pink, 2020; Raabe et al., 2019; Zingora et al., 2019). However, an overlap in social categories might result in spurious conclusions about who influences whom. For example, same-gender peers might also include same-ethnic peers. In a situation when students are influenced by same-gender peers but not necessarily same-ethnic peers, same-ethnic peers would be shown to exert normative pressure on adolescents since this group includes same-gender peers who do exert social influence. We hope that future research will address this issue and provide more guidance on the selection of potentially important social categories for peer influence.

We believe that a method to investigate normative pressure and friendship influence simultaneously using longitudinal social network analysis opens interesting avenues for the investigation of an interplay between these types of peer influence. For example, as already suggested in the introduction, students could navigate friendship selection to escape undesirable normative pressure. Future research could look closely at the complex consequences of the mutual impact of normative pressure and friendship influence on the spread of attitudes and behaviors.

Finally, we encourage testing our conclusions in novel settings. Our study was conducted with data from one national context—Germany—and focuses on the setting of offline social relationships in schools. Future work could explore whether our findings also apply to different national contexts and to workplace or online settings.

Our intention in this research was to bring robust evidence about the importance of different types of peer influence in developing attitudes and behaviors. The results were robust since the development of all studied outcomes—academic drive, prejudice, and religiosity—was linked to the same types of peer influence. However, one limitation is that we used proxies of these outcomes. This applies particularly to religiosity since different religions could have different customs about the frequency of visiting a place of worship. One task of future research could be to investigate normative pressure for different religious groups.

## Conclusion

Peer influence plays a pivotal role in the development of adolescents. It is of paramount importance to reliably capture the different forms of peer influence, as they have different implications for intervention strategies relying on peer influence. We improved the understanding of peer influence and underscored the critical value of normative pressure. Capturing normative pressure is important for studying questions such as who influences whom, who are especially influential students in schools, or how attitudes and behaviors spread in schools. We developed a novel approach that could be adopted by other scientists, drawing on the benefits of social network science. Our research indicates that students were influenced by same-group peers via normative pressure and by friends via friendship influence, especially those friends of the same gender as the students.

## Supplemental Material

sj-docx-1-psp-10.1177_01461672251366388 – Supplemental material for Effects of Friendship Influence and Normative Pressure on Adolescent Attitudes and Behaviors Supplemental material, sj-docx-1-psp-10.1177_01461672251366388 for Effects of Friendship Influence and Normative Pressure on Adolescent Attitudes and Behaviors by Tibor Zingora and Andreas Flache in Personality and Social Psychology Bulletin

## Notes

1. We want to thank an anonymous reviewer for pointing us toward this hypothesis.

## References

[bibr1-01461672251366388] Badham J. Kee F. Hunter R. F. (2019). Effectiveness variation in simulated school-based network interventions. Applied Network Science, 4(1), 70. 10.1007/s41109-019-0168-6

[bibr2-01461672251366388] Bandura A. (1977). Social learning theory. Prentice-Hall.

[bibr3-01461672251366388] Berndt T. J. (1996). Transitions in friendship and friends’ influence. In Graber J. A. Brooks-Gunn J. Petersen A. C. (Eds.), Transitions through adolescence: Interpersonal domains and context (pp. 57–84). Lawrence Erlbaum Associates, Inc.

[bibr4-01461672251366388] Boda Z. (2018). Social influence on observed race. Sociological Science, 5, 29–57. 10.15195/v5.a3

[bibr5-01461672251366388] Boda Z. Elmer T. Vörös A. Stadtfeld C. (2020). Short-term and long-term effects of a social network intervention on friendships among university students. Scientific Reports, 10(1), 2889. 10.1038/s41598-020-59594-z PMC7031228 32076003

[bibr6-01461672251366388] Bonacich P. (1987). Power and centrality: A family of measures. American Journal of Sociology, 92(5), 1170–1182. 10.1086/228631

[bibr7-01461672251366388] Bonacich P. (2007). Some unique properties of eigenvector centrality. Social Networks, 29(4), 555–564. 10.1016/j.socnet.2007.04.002

[bibr8-01461672251366388] Borgatti S. P. (2005). Centrality and network flow. Social Networks, 27(1), 55–71. 10.1016/j.socnet.2004.11.008

[bibr9-01461672251366388] Bracegirdle C. Reimer N. K. van Zalk M. Hewstone M. Wölfer R. (2022). Disentangling contact and socialization effects on outgroup attitudes in diverse friendship networks. Journal of Personality and Social Psychology, 122(1), 1–15. 10.1037/pspa0000240 34516182

[bibr10-01461672251366388] Campbell R. Starkey F. Holliday J. Audrey S. Bloor M. Parry-Langdon N. Hughes R. Moore L. (2008). An informal school-based peer-led intervention for smoking prevention in adolescence (ASSIST): A cluster randomised trial. The Lancet, 371(9624), 1595–1602. 10.1016/S0140-6736(08)60692-3 PMC2387195 18468543

[bibr11-01461672251366388] Cialdini R. B. Kallgren C. A. Reno R. R. (1991). A focus theory of normative conduct: A theoretical refinement and reevaluation of the role of norms in human behavior. Advances in Experimental Social Psychology, 24, 201–234. 10.1016/S0065-2601(08)60330-5

[bibr12-01461672251366388] DeLay D. Burk W. J. Laursen B. (2022). Assessing peer influence and susceptibility to peer influence using individual and dyadic moderators in a social network context: The case of adolescent alcohol misuse. International Journal of Behavioral Development, 46(3), 208–221. 10.1177/01650254221084102 35645435 PMC9139630

[bibr13-01461672251366388] Dijkstra J. K. Lindenberg S. Veenstra R. (2008). Beyond the class norm: Bullying behavior of popular adolescents and its relation to peer acceptance and rejection. Journal of Abnormal Child Psychology, 36(8), 1289. 10.1007/s10802-008-9251-7 18607717

[bibr14-01461672251366388] Festinger L. (1954). A theory of social comparison processes. Human Relations, 7(2), 117–140.

[bibr15-01461672251366388] Flache A. (2002). The rational weakness of strong ties: Failure of group solidarity in a highly cohesive group of rational agents. The Journal of Mathematical Sociology, 26(3), 189–216. 10.1080/00222500212988

[bibr16-01461672251366388] Flache A. Macy M. W. (1996). The weakness of strong ties: Collective action failure in a highly cohesive group. Journal of Mathematical Sociology, 21(1–2), 3–28.

[bibr17-01461672251366388] Geven S. Weesie J. van Tubergen F. (2013). The influence of friends on adolescents’ behavior problems at school: The role of ego, alter and dyadic characteristics. Social Networks, 35(4), 583–592. 10.1016/j.socnet.2013.08.002

[bibr18-01461672251366388] Giletta M. Choukas-Bradley S. Maes M. Linthicum K. P. Card N. A. Prinstein M. J. (2021). A meta-analysis of longitudinal peer influence effects in childhood and adolescence. Psychological Bulletin, 147(7), 719–747. 10.1037/bul0000329 34855429

[bibr19-01461672251366388] Gremmen M. C. Berger C. Ryan A. M. Steglich C. E. G. Veenstra R. Dijkstra J. K. (2019). Adolescents’ friendships, academic achievement, and risk behaviors: Same-behavior and cross-behavior selection and influence processes. Child Development, 90, e192–e211. 10.1111/cdev.13045 29450883

[bibr20-01461672251366388] Hogg M. A. Reid S. A. (2006). Social identity, self-categorization, and the communication of group norms. Communication Theory, 16(1), 7–30. 10.1111/j.1468-2885.2006.00003.x

[bibr21-01461672251366388] Jetten J. Hornsey M. J. (2014). Deviance and dissent in groups. Annual Review of Psychology, 65(1), 461–485. 10.1146/annurev-psych-010213-115151 23751035

[bibr22-01461672251366388] Knapp G. Hartung J. (2003). Improved tests for a random effects meta-regression with a single covariate. Statistics in Medicine, 22(17), 2693–2710. 10.1002/sim.1482 12939780

[bibr23-01461672251366388] Krause R. Iashina A. (2019, September 13). Multiple imputation for RSiena – Network and behavior. https://www.stats.ox.ac.uk/~snijders/siena/MultipleImputationNetworkAndBehavior.html

[bibr24-01461672251366388] Krause R. Huisman M. Snijders T. A. B. (2018). Multiple imputation for longitudinal network data. Italian Journal of Applied Statistics, 30(1), 33–57. 10.26398/IJAS.0030-002

[bibr25-01461672251366388] Laninga-Wijnen L. Steglich C. E. G. Harakeh Z. Vollebergh W. Veenstra R. Dijkstra J. K. (2019). The role of prosocial and aggressive popularity norm combinations in prosocial and aggressive friendship processes. Journal of Youth and Adolescence, 49, 645–663. 10.1007/s10964-019-01088-x 31407189 PMC7079708

[bibr26-01461672251366388] Leszczensky L. Flache A. Sauter L. (2020). Does the share of religious ingroup members affect how important religion is to adolescents? Applying Optimal Distinctiveness Theory to four European countries. Journal of Ethnic and Migration Studies, 46(17), 3703–3721. 10.1080/1369183X.2019.1620419

[bibr27-01461672251366388] Leszczensky L. Pink S. (2020). Are birds of a feather praying together? Assessing friends’ influence on Muslim youth’ religiosity in German. Social Psychology Quarterly, 83, 251–271. 10.1177/0190272520936633

[bibr28-01461672251366388] Leszczensky L. Pink S. Kretschmer D. Kalter F. (2020). Friendship and Identity in School (Version 1.0.0.) [Dataset]. German Center for Intergration and Migration Research (DeZIM). 10.34882/dezim.fis.c.1.0.0.

[bibr29-01461672251366388] Leszczensky L. Stark T. H. Flache A. Munniksma A. (2016). Disentangling the relation between young immigrants’ host country identification and their friendships with natives. Social Networks, 44, 179–189. 10.1016/j.socnet.2015.08.001

[bibr30-01461672251366388] Levine T. R. Wheeless L. R. (1997). Situational intimacy as a predictor of compliance-gaining tactic selection. Communication Research Reports, 14(2), 132–144. 10.1080/08824099709388655

[bibr31-01461672251366388] Li S. Krackhardt D. Niezink N. M. D. (2023). Do your friends stress you out? A field study of the spread of stress through a community network. Journal of Personality and Social Psychology, 125(1), 100–116. 10.1037/pspi0000415 36633994

[bibr32-01461672251366388] McPherson M. Smith-Lovin L. Cook J. M. (2001). Birds of a Feather: Homophily in Social Networks. Annual Review of Sociology, 27(1), 415–444. 10.1109/CFIS.2015.7391644

[bibr33-01461672251366388] Newcomb A. F. Bagwell C. L. (1995). Children’s friendship relations: A meta-analytic review. Psychological Bulletin, 117(2), 306–347. 10.1037//0033-2909.117.2.306

[bibr34-01461672251366388] Paluck E. L. Shepherd H. (2012). The salience of social referents: A field experiment on collective norms and harassment behavior in a school social network. Journal of Personality and Social Psychology, 103(6), 899–915. 10.1037/a0030015 22984831

[bibr35-01461672251366388] Paluck E. L. Shepherd H. Aronow P. M. (2016). Changing climates of conflict: A social network experiment in 56 schools. Proceedings of the National Academy of Sciences of the United States of America, 113(15), 566–571. 10.1073/pnas.1514483113 26729884 PMC4725542

[bibr36-01461672251366388] Perkins H. W. Craig D. W. Perkins J. M. (2011). Using social norms to reduce bullying: A research intervention among adolescents in five middle schools. Group Processes & Intergroup Relations, 14(5), 703–722. 10.1177/1368430210398004

[bibr37-01461672251366388] Raabe I. J. Boda Z. Stadtfeld C. (2019). The social pipeline: How friend influence and peer exposure widen the STEM gender gap. Sociology of Education, 92(2), 105–123. 10.1177/0038040718824095

[bibr38-01461672251366388] Ripley R. M. Snijders T. A. B. Boda Z. Vörös A. Preciado P. (2024, September 3). Manual for RSiena. https://www.stats.ox.ac.uk/~snijders/siena/RSiena_Manual.pdf

[bibr39-01461672251366388] Schaefer D. R. Adams J. Haas S. A. (2013). Social networks and smoking: Exploring the effects of peer influence and smoker popularity through simulations. Health Education and Behavior, 40, 24S–32S. 10.1177/1090198113493091 PMC4587387 24084397

[bibr40-01461672251366388] Snijders T. A. B. Baerveldt C. (2003). A multilevel network study of the effects of delinquent behavior on friendship. Journal of Mathematical Sociology, 27, 123–151.

[bibr41-01461672251366388] Snijders T. A. B. van de Bunt G. G. Steglich C. E. G. (2010). Introduction to stochastic actor-based models for network dynamics. Social Networks, 32(1), 44–60. 10.1016/j.socnet.2009.02.004

[bibr42-01461672251366388] Stadtfeld C. Snijders T. A. B. Steglich C. E. G. Van Duijn M. (2020). Statistical Power in Longitudinal Network Studies. Sociological Methods & Research, 49(4), 1103–1132. 10.1177/0049124118769113

[bibr43-01461672251366388] Stark T. H. (2015). Understanding the selection bias social network processes and the effect of prejudice on the avoidance of outgroup friends. Social Psychology Quarterly, 78(2), 127–150. 10.1177/0190272514565252

[bibr44-01461672251366388] Steglich C. E. G. Snijders T. A. B. Pearson M. (2010). Dynamic networks and behavior: separating selection from influence. Sociological Methodology, 40(1), 329–393. 10.1111/j.1467-9531.2010.01225.x

[bibr45-01461672251366388] Steglich C. E. G. Snijders T. A. B. West P. (2006). Applying SIENA. Methodology, 2(1), 48–56. 10.1027/1614-2241.2.1.48

[bibr46-01461672251366388] Tajfel H. (1982). Social psychology of intergroup relations. Annual Review of Psychology, 33(1), 1–39. 10.1146/annurev.ps.33.020182.000245

[bibr47-01461672251366388] Tajfel H. Turner J. C. Austin W. G. Worchel S. (1979). An integrative theory of intergroup conflict. In Austin W. G. Worchel S. (Eds.), The social psychology of intergroup relations (pp. 33–37). Brooks/Cole.

[bibr48-01461672251366388] Turner J. C. Hogg M. A. Oakes P. J. Reicher S. D. Wetherell M. S. (1987). Rediscovering the social group: A self-categorization theory. Basil Blackwell.

[bibr49-01461672251366388] Valente T. W. (2012). Network interventions. Science, 337(6090), 49–53. 10.1126/science.1217330 22767921

[bibr50-01461672251366388] van Buuren S. Groothuis-Oudshoorn K. (2011). mice: Multivariate Imputation by Chained Equations in R. Journal of Statistical Software, 45(3), 1–67. 10.18637/jss.v045.i03

[bibr51-01461672251366388] Van Zalk M. H. W. Kerr M. Van Zalk N. Stattin H . (2013). Xenophobia and tolerance toward immigrants in adolescence: Cross-influence processes within friendships. Journal of Abnormal Child Psychology, 41, 627–639. 10.1007/s10802-012-9694-8 23129524

[bibr52-01461672251366388] Veenstra R. Dijkstra J. K. Steglich C. E. G. Van Zalk M. H. W. (2013). Network–behavior dynamics. Journal of Research on Adolescence, 23(3), 399–412. 10.1111/jora.12070

[bibr53-01461672251366388] Veenstra R. Laninga-Wijnen L. (2022). Peer network studies and interventions in adolescence. Current Opinion in Psychology, 44, 157–163. 10.1016/j.copsyc.2021.09.015 34662775

[bibr54-01461672251366388] Viechtbauer W. (2010). Conducting meta-analyses in R with the metafor Package. Journal of Statistical Software, 36, 1–48. 10.18637/jss.v036.i03

[bibr55-01461672251366388] Weerman F. M. (2011). Delinquent peers in context: A longitudinal network analysis of selection and influence effects. Criminology, 49(1), 253–286. 10.1111/j.1745-9125.2010.00223.x

[bibr56-01461672251366388] Wölfer R. Hewstone M. (2017). Beyond the dyadic perspective: 10 Reasons for using social network analysis in intergroup contact research. British Journal of Social Psychology, 56(3), 609–617. 10.1111/bjso.12195 28332293

[bibr57-01461672251366388] Xu J. (2005). Purposes for doing homework reported by middle and high school students. The Journal of Educational Research, 99(1), 46–55. 10.3200/JOER.99.1.46-55

[bibr58-01461672251366388] Zingora T. (2024). On the spurious effect of intergroup friendship on outgroup attitudes in schools: The role of social influence and the positive impact of exposure to outgroup peers. British Journal of Social Psychology, 64, 12797. 10.1111/bjso.12797 PMC11923959 39291422

[bibr59-01461672251366388] Zingora T. Stark T. H. Flache A. (2019). Who is most influential? Adolescents’ intergroup attitudes and peer influence within a social network. Group Processes & Intergroup Relations, 23(5), 684–709. 10.1177/1368430219869460

