# Supplementary material for: Effects of Friendship Influence and Normative Pressure on Adolescent Attitudes and Behaviors
Source: Pers Soc Psychol Bull. 2025 Sep 25;52(10):3216–34. doi: 10.1177/01461672251366388 (PMC13507506; doi:10.1177/01461672251366388)
Supplement: Supplementary file 1 [file sj-docx-1-psp-10.1177_01461672251366388.docx]

Appendix to

**Effects of friendship influence and normative pressure on adolescent attitudes and behaviors**

**Figure 1. Ethnic groups**

**Table 1**

*Technical description of effects, including SIENA terminology*

| **Effect** | **Effects as labeled in the article** | **RSIENA terminology** | **Explanation** |
| --- | --- | --- | --- |
|  |  |  |  |
| Alter’s total of dyadic covariate W | Number of [group] friends* | totWAlt | Tendency for target adolescents with more [group] friends to develop more positive attitudes towards a secondary outgroup. |
| Average similarity weighted by dyadic covariate | Social influence of [group] friends* | totSimW | Tendency for target adolescents to adopt an average attitude of their [group] friends |
| Main covariate effect | The number of [group] classmates* | effFrom | An effect of the number of [group] classmates on the development of secondary outgroup attitudes |
|  | The number of classmates | effFrom | An effect of the number of classmates on the development of secondary outgroup attitudes |
| Outdegree (density) | Density | density | Tendency to form new friendship ties |
| Reciprocity | Reciprocity | recip | Tendency to reciprocate friendship ties |
| Geometrically weighted edgewise shared partners | Geometrically weighted edgewise shared partners | gwespFF | Tendency to befriend the friends of friends (transitive) |
| Geometrically weighted edgewise shared partners | Geometrically weighted edgewise shared partners | gwespRR | Tendency to befriend the friends of friends given that ties between friends are reciprocal |
| Geometrically weighted edgewise shared partners | Geometrically weighted edgewise shared partners | gwespBB | Tendency to receive friendship nominations from friends of friends |
| In-degree related popularity effect squared | Indegree popularity squared | inPopSqrt | Tendency of adolescents to nominate friends who have already had many friendship nominations squared |
| Out-degree related popularity effect squared | Outdegree popularity squared | outPopSqrt | Tendency of adolescents to nominate friends who nominate many friends squared |
| Covariate-ego or covariate-related activity | Gender / [outcome]ego | egoX | A tendency of adolescents to nominate more peers as friends depending on adolescents’ values of a covariate |
| Covariate-alter or covariate-related popularity | Gender / [outcome]alter | altX | A tendency of adolescents to prefer friends with higher values of a covariate |
| Same covariate (or covariate-related identity) | Gender / ethnic homophily | sameX | A preference for friends with the same value on a covariate |
| Similar covariate (or covariate-related identity) | Outcome homophily | simX | A preference for friends with similar outcome value |
| Group average | Group average of [outcome] | avGroup | Effect of an average mean of an outcome in the network on an outcome of an adolescent. This effect can express regression to the mean. |
| Average similarity | Friendship influence | avSim | Tendency for target adolescents to adopt an average [outcome]of their friends |
| Average similarity weighted by dyadic covariate | Friendship influence of [ingroup / outgroup] friends | avSimW | Tendency for target adolescents to adopt an average attitude of their [ingroup / outgroup] friends |
| Main covariate effect | Gender | effFrom | The effect of gender on the development of [outcome] |
| Average similarity | Normative pressure | avSim | Tendency for target adolescents to adopt an average [outcome] of peers with the same covariate, i.e., gender or ethnicity. |
| Linear shape | Linear shape | linear | Potential trends in the data toward lower or  higher values on the dependent variable |
| Quadratic shape | Quadratic shape | quad | The tendency of adolescents with very  positive or very negative attitudes to develop  even more extreme attitudes |

*Note.* Group represents, for example, ingroup or primary outgroup friends.

**Goodnes of fit**

The goodness of fit was assessed in terms of accurately captured indegree, outdegree, and geodesic distribition as well as triad census (Ripley et al., 2024). We report p-values of these tests. P-values that differ from zero indicate that real data characteristics were similar to those simulated based on our model specifications. For this purpose, we estimated one model for all school grades. As we were interested in this test whether we captures the nework evolution, it was sufficient to estimate one model as this model was similar for all models reported in the paper. Using this model, we assessed the goodnes of fit for all school grades seprately and combined p-value. Our model yielded good model fit. Specifically, p-values were as follows: indegree distribution = .188, outdegree distribution = .036, geodesic distribution = .188, triadic census = .024.

**Proportion of missing values**

Some attributes, such as gender and ethnicity, were constant across waves, allowing us to utilize information from different waves of data. This is not possible for outcomes, which were measured at each wave and thus had higher missingness. For instance, about 10% of gender and ethnicity values were imputed, compared to roughly 57% of outcome values. However, many of these missing cells result from the network structure including students who were not present at a specific wave. When focusing only on outcome data, 36.6% of participants did not report any outcome at T1, 28.7% at T2, and 27.1% at T3. Across all waves, 10.7% of participants had no outcome data at any time point.

**Table 2**

Complete SAOM results – academic drive as an outcome and group membership based on gender

|  | Model 1 | | | Model 2 | | | Model 3 | | | Model 4 | | | Model 5 | | |
| --- | --- | --- | --- | --- | --- | --- | --- | --- | --- | --- | --- | --- | --- | --- | --- |
|  | Estimate | SE | N | Estimate | SE | N | Estimate | SE | N | Estimate | SE | N | Estimate | SE | N |
| **Behavioral Function** |  |  |  |  |  |  |  |  |  |  |  |  |  |  |  |
| ***Normative pressure*** |  |  |  |  |  |  |  |  |  |  |  |  |  |  |  |
| Normative pressure  same-sex peers |  |  |  | 4.63^***^ | .58 | 29 | 3.05^***^ | .76 | 28 |  |  |  | 3.06^***^ | .69 | 26 |
| ***Friendship influence*** |  |  |  |  |  |  |  |  |  |  |  |  |  |  |  |
| Friendship influence | 3.14^***^ | .36 | 29 |  |  |  | 2.89^***^ | .40 | 28 |  |  |  |  |  |  |
| Friendship influence  same-gender peers |  |  |  |  |  |  |  |  |  | 2.84^***^ | .40 | 27 | 2.18^***^ | .43 | 26 |
| Friendship influence  different-gender peers |  |  |  |  |  |  |  |  |  | .65 | .73 | 25 | .95 | .76 | 23 |
|  |  |  |  |  |  |  |  |  |  |  |  |  |  |  |  |
| ***Other effects*** |  |  |  |  |  |  |  |  |  |  |  |  |  |  |  |
| Rate 1 | 2.42^***^ | .20 | 29 | 2.50^***^ | .20 | 29 | 2.46^***^ | .20 | 28 | 2.39^***^ | .20 | 27 | 2.37^***^ | .19 | 26 |
| Rate 2 | 2.22^***^ | .16 | 29 | 2.22^***^ | .15 | 29 | 2.29^***^ | .16 | 28 | 2.18^***^ | .16 | 27 | 2.19^***^ | .26 | 26 |
| Linear shape | -.31 | .16 | 29 | -.12^*^ | .05 | 29 | -.13^*^ | .06 | 28 | -.29^*^ | .14 | 27 | -.16 | .05 | 26 |
| Quadratic shape | .02 | .02 | 29 | .13^***^ | .04 | 29 | .16^***^ | .04 | 28 | .002 | .03 | 27 | .14^**^ | .05 | 26 |
| School-grade average | -.32 | .35 | 25 | -.79^*^ | .37 | 24 | -.86^*^ | .39 | 25 | -.40 | .37 | 23 | -.98^*^ | .45 | 22 |
| Being male vs. female | -.12^***^ | .03 | 29 | -.03 | .03 | 29 | -.04 | .04 | 28 | -.10 | .04^*^ | 27 | -.04 | .04 | 26 |
| **Network function** |  |  |  |  |  |  |  |  |  |  |  |  |  |  |  |
| Rate 1 | 12.66^***^ | .54 | 29 | 12.63^***^ | .54 | 29 | 12.78^***^ | .56 | 28 | 12.63^***^ | .56 | 27 | 12.77^***^ | .58 | 26 |
| Rate 2 | 11.93^***^ | .48 | 29 | 11.95^***^ | .47 | 29 | 11.94^***^ | .48 | 28 | 11.91^***^ | .50 | 27 | 11.90^***^ | .50 | 26 |
| Density | -1.84^***^ | .06 | 29 | -1.85^***^ | .06 | 29 | -1.85^***^ | .06 | 28 | -1.87^***^ | .06 | 27 | -1.84^***^ | .06 | 26 |
| Reciprocity | 1.65^***^ | .05 | 29 | 1.65^***^ | .05 | 29 | 1.65^***^ | .05 | 28 | 1.65^***^ | .05 | 27 | 1.65^***^ | .05 | 26 |
| GWESPFF | 1.90^***^ | .05 | 29 | 1.90^***^ | .05 | 29 | 1.89^***^ | .05 | 28 | 1.91^***^ | .05 | 27 | 1.90^***^ | .05 | 26 |
| GWESPBB | .69^***^ | .04 | 29 | .70^***^ | .04 | 29 | .70^***^ | .04 | 28 | .70^***^ | .04 | 27 | .69^***^ | .04 | 26 |
| GWESPRR | -1.03^***^ | .07 | 29 | -1.02^***^ | .07 | 29 | -1.02^***^ | .07 | 28 | -1.04^***^ | .07 | 27 | -1.02^***^ | .07 | 26 |
| Indegree popularity squared | -.19^***^ | .02 | 29 | -.18^***^ | .02 | 29 | -.18^***^ | .02 | 28 | -.18^***^ | .02 | 27 | -.19^***^ | .02 | 26 |
| Outdegree popularity squared | -.60^***^ | .02 | 29 | -.60^***^ | .02 | 29 | -.60^***^ | .02 | 28 | -.60^***^ | .02 | 27 | -.60^***^ | .03 | 26 |
| Gender alter | .001 | .02 | 29 | .001 | .02 | 29 | -.001 | .02 | 28 | -.003 | .02 | 27 | -.003 | .02 | 26 |
| Gender ego | .001 | .03 | 29 | -.001 | .03 | 29 | -.003 | .03 | 28 | .01 | .03 | 27 | .01 | .03 | 26 |
| Same gender | .36^***^ | .02 | 29 | .36^***^ | .02 | 29 | .36^***^ | .02 | 28 | .36^***^ | .02 | 27 | .37^***^ | .02 | 26 |
| Same ethnicity | .18^***^ | .02 | 29 | .18^***^ | .02 | 29 | .18^***^ | .02 | 28 | .18^***^ | .02 | 27 | .19^***^ | .02 | 26 |
| Academic drive alter | .03 | .02 | 29 | .03 | .02 | 29 | .03 | .02 | 28 | .03 | .02 | 27 | .02 | .02 | 26 |
| Academic drive ego | -.16^***^ | .04 | 29 | -.16^***^ | .04 | 29 | -.16^***^ | .04 | 28 | -.16^***^ | .04 | 27 | -.16^***^ | .04 | 26 |
| Similar academic drive | -.06 | .16 | 29 | -.03 | .15 | 29 | -.06 | .15 | 28 | -.04 | .16 | 27 | -.07 | .16 | 26 |
|  |  |  |  |  |  |  |  |  |  |  |  |  |  |  |  |

*Note.* Model 1 is idendical regardless of whether group membership is based on gender or ethnicity.

**Table 3**

Complete SAOM results – academic drive as an outcome and group membership based on ethnicity

|  | Model 1 | | | Model 2 | | | Model 3 | | | Model 4 | | | Model 5 | | |
| --- | --- | --- | --- | --- | --- | --- | --- | --- | --- | --- | --- | --- | --- | --- | --- |
|  | Estimate | SE | N | Estimate | SE | N | Estimate | SE | N | Estimate | SE | N | Estimate | SE | N |
| **Behavioral Function** |  |  |  |  |  |  |  |  |  |  |  |  |  |  |  |
| ***Normative pressure*** |  |  |  |  |  |  |  |  |  |  |  |  |  |  |  |
| Normative pressure  same-sex peers |  |  |  | 1.14^***^ | .23 | 29 | .96^***^ | .26 | 29 |  |  |  | .82^**^ | .28 | 28 |
| ***Friendship influence*** |  |  |  |  |  |  |  |  |  |  |  |  |  |  |  |
| Friendship influence | 3.14^***^ | .36 | 29 |  |  |  | 3.10^***^ | .37 | 29 |  |  |  |  |  |  |
| Friendship influence  same-gender peers |  |  |  |  |  |  |  |  |  | 1.13^***^ | .34 | 28 | .88^*^ | .36 | 28 |
| Friendship influence  different-gender peers |  |  |  |  |  |  |  |  |  | 2.59^***^ | .40 | 28 | 2.61^***^ | .41 | 28 |
|  |  |  |  |  |  |  |  |  |  |  |  |  |  |  |  |
| ***Other effects*** |  |  |  |  |  |  |  |  |  |  |  |  |  |  |  |
| Rate 1 | 2.42^***^ | .20 | 29 | 2.42^***^ | .19 | 28 | 2.45^***^ | .20 | 28 | 2.40^***^ | .20 | 28 | 2.45^***^ | .20 | 28 |
| Rate 2 | 2.22^***^ | .16 | 29 | 2.14^***^ | .15 | 29 | 2.21^***^ | .15 | 29 | 2.19^***^ | .16 | 27 | 2.26^***^ | .17 | 28 |
| Linear shape | -.31 | .16 | 29 | -.37^**^ | .13 | 29 | -.15^***^ | .05 | 29 | -.16^**^ | .05 | 27 | -.24^***^ | .07 | 28 |
| Quadratic shape | .02 | .02 | 29 | -.08^**^ | .03 | 29 | .08^**^ | .03 | 29 | -.01 | .03 | 28 | .06^*^ | .03 | 28 |
| School-grade average | -.32 | .35 | 25 | -.15 | .32 | 25 | -.51 | .36 | 24 | -.38 | .36 | 24 | -.58 | .40 | 28 |
| Being male vs. female | -.12^***^ | .03 | 29 | -.15^***^ | .03 | 29 | -.11^***^ | .03 | 29 | -.12^***^ | .03 | 28 | -.13^***^ | .04 | 28 |
| **Network function** |  |  |  |  |  |  |  |  |  |  |  |  |  |  |  |
| Rate 1 | 12.65^***^ | .54 | 29 | 12.67^***^ | .54 | 29 | 12.70^***^ | .55 | 29 | 12.76^***^ | .55 | 28 | 12.67^***^ | .55 | 28 |
| Rate 2 | 11.93^***^ | .48 | 29 | 11.95^***^ | .47 | 29 | 11.97^***^ | .48 | 29 | 11.91^***^ | .48 | 28 | 11.89^***^ | .48 | 28 |
| Density | -1.84^***^ | .06 | 29 | -1.85^***^ | .06 | 29 | -1.84^***^ | .06 | 29 | -1.85^***^ | .06 | 28 | -1.85^***^ | .06 | 28 |
| Reciprocity | 1.65^***^ | .05 | 29 | 1.65^***^ | .05 | 29 | 1.65^***^ | .05 | 29 | 1.65^***^ | .05 | 28 | 1.65^***^ | .05 | 28 |
| GWESPFF | 1.90^***^ | .05 | 29 | 1.90^***^ | .05 | 29 | 1.89^***^ | .05 | 29 | 1.90^***^ | .05 | 28 | 1.91^***^ | .05 | 28 |
| GWESPBB | .69^***^ | .04 | 29 | .69^***^ | .04 | 29 | .69^***^ | .04 | 29 | .69^***^ | .04 | 28 | .70^***^ | .04 | 28 |
| GWESPRR | -1.02^***^ | .07 | 29 | -1.02^***^ | .07 | 29 | -1.01^***^ | .07 | 29 | -1.02^***^ | .07 | 28 | -1.04^***^ | .06 | 28 |
| Indegree popularity squared | -.19^***^ | .02 | 29 | -.19^***^ | .02 | 29 | -.18^***^ | .02 | 29 | -.19^***^ | .02 | 28 | -.19^***^ | .02 | 28 |
| Outdegree popularity squared | -.60^***^ | .02 | 29 | -.60^***^ | .02 | 29 | -.60^***^ | .02 | 29 | -.60^***^ | .02 | 28 | -.60^***^ | .02 | 28 |
| Gender alter | .001 | .02 | 29 | -.001 | .02 | 29 | -.002 | .02 | 29 | -.001 | .02 | 28 | -.001 | .02 | 28 |
| Gender ego | .001 | .03 | 29 | .0002 | .03 | 29 | .001 | .03 | 29 | .005 | .03 | 28 | -.001 | .03 | 28 |
| Same gender | .36^***^ | .02 | 29 | .36^***^ | .02 | 29 | .37^***^ | .02 | 29 | .37^***^ | .02 | 28 | .36^***^ | .02 | 28 |
| Same ethnicity | .18^***^ | .02 | 29 | .19^***^ | .02 | 29 | .18^***^ | .02 | 29 | .18^***^ | .02 | 28 | .19^***^ | .02 | 28 |
| Academic drive alter | .03 | .02 | 29 | .03 | .02 | 29 | .03 | .02 | 29 | .03 | .02 | 28 | .03 | .02 | 28 |
| Academic drive ego | -.16^***^ | .04 | 29 | -.16^***^ | .04 | 29 | -.16^***^ | .04 | 29 | -.16^***^ | .04 | 28 | -.17^***^ | .04 | 28 |
| Similar academic drive | -.06 | .16 | 29 | -.05 | .15 | 29 | -.06 | .15 | 28 | -.09 | .16 | 27 | -.08 | .16 | 28 |
|  |  |  |  |  |  |  |  |  |  |  |  |  |  |  |  |

*Note.* Model 1 is idendical regardless of whether group membership is based on gender or ethnicity.

**Table 4**

Complete SAOM results – prejudice as an outcome and group membership based on gender

|  | Model 1 | | | Model 2 | | | Model 3 | | | Model 4 | | | Model 5 | | |
| --- | --- | --- | --- | --- | --- | --- | --- | --- | --- | --- | --- | --- | --- | --- | --- |
|  | Estimate | SE | N | Estimate | SE | N | Estimate | SE | N | Estimate | SE | N | Estimate | SE | N |
| **Behavioral Function** |  |  |  |  |  |  |  |  |  |  |  |  |  |  |  |
| ***Normative pressure*** |  |  |  |  |  |  |  |  |  |  |  |  |  |  |  |
| Normative pressure  same-sex peers |  |  |  | 2.94^***^ | .58 | 26 | 2.38^***^ | .64 | 26 |  |  |  | 2.41^***^ | .74 | 25 |
| ***Friendship influence*** |  |  |  |  |  |  |  |  |  |  |  |  |  |  |  |
| Friendship influence | 1.01^***^ | .29 | 27 |  |  |  | .92^***^ | .31 | 26 |  |  |  |  |  |  |
| Friendship influence  same-gender peers |  |  |  |  |  |  |  |  |  | .99^***^ | .29 | 26 | .86^**^ | .34 | 25 |
| Friendship influence  different-gender peers |  |  |  |  |  |  |  |  |  | -.23 | .52 | 22 | -.06 | .52 | 25 |
|  |  |  |  |  |  |  |  |  |  |  |  |  |  |  |  |
| ***Other effects*** |  |  |  |  |  |  |  |  |  |  |  |  |  |  |  |
| Rate 1 | 3.81^***^ | .27 | 27 | 3.75^***^ | .27 | 26 | 3.76^***^ | .26 | 26 | 3.71^***^ | .28 | 26 | 3.76^***^ | .27 | 25 |
| Rate 2 | 3.06^***^ | .25 | 27 | 2.95^***^ | .22 | 26 | 2.99^***^ | .22 | 26 | 2.96^***^ | .24 | 26 | 3.04^***^ | .26 | 25 |
| Linear shape | .22 | .38 | 26 | .64 | .48 | 26 | .68^*^ | .34 | 26 | .17 | .11 | 26 | .28^*^ | .13 | 25 |
| Quadratic shape | -.01 | .02 | 27 | .11 | .04^**^ | 26 | .11^**^ | .04 | 26 | -.02 | .03 | 26 | .11^*^ | .05 | 25 |
| School-grade average | -.16 | .21 | 27 | -.32 | .24 | 26 | -.38 | .26 | 25 | -.12 | .22 | 24 | -.27 | .29 | 23 |
| Being male vs. female | -.04 | .02 | 27 | .02 | .03 | 26 | .01 | .03 | 26 | -.04 | .03 | 26 | .004 | .03 | 25 |
| **Network function** |  |  | 27 |  |  |  |  |  | 26 |  |  | 26 |  |  | 25 |
| Rate 1 | 12.63^***^ | .52 | 27 | 12.72^***^ | .53 | 26 | 12.66^***^ | .54 | 26 | 12.68^***^ | .54 | 26 | 12.64^***^ | .56 | 25 |
| Rate 2 | 12.02^***^ | .47 | 27 | 12.23^***^ | .46 | 26 | 12.22^***^ | .46 | 26 | 12.18^***^ | .46 | 26 | 12.15^***^ | .47 | 25 |
| Density | -1.85^***^ | .05 | 27 | -1.85^***^ | .06 | 26 | -1.85^***^ | .06 | 26 | -1.85^***^ | .06 | 26 | -1.87^***^ | .06 | 25 |
| Reciprocity | 1.66^***^ | .05 | 27 | 1.65^***^ | .05 | 26 | 1.65^***^ | .05 | 26 | 1.65^***^ | .05 | 26 | 1.65^***^ | .05 | 25 |
| GWESPFF | 1.92^***^ | .05 | 27 | 1.93^***^ | .05 | 26 | 1.93^***^ | .05 | 26 | 1.94^***^ | .05 | 26 | 1.93^***^ | .05 | 25 |
| GWESPBB | .69^***^ | .04 | 27 | .69^***^ | .04 | 26 | .69^***^ | .04 | 26 | .69^***^ | .04 | 26 | .70^***^ | .03 | 25 |
| GWESPRR | -1.02^***^ | .07 | 27 | -1.03^***^ | .07 | 26 | -1.04^***^ | .07 | 26 | -1.04^***^ | .07 | 26 | -1.05^***^ | .07 | 25 |
| Indegree popularity squared | -.17^***^ | .02 | 27 | -.17^***^ | .02 | 26 | -.17^***^ | .02 | 26 | -.18^***^ | .02 | 26 | -.16^***^ | .02 | 25 |
| Outdegree popularity squared | -.59^***^ | .02 | 27 | -.59^***^ | .02 | 26 | -.59^***^ | .02 | 26 | -.59^***^ | .02 | 26 | -.60^***^ | .03 | 25 |
| Gender alter | .005 | .03 | 27 | .001 | .02 | 26 | .001 | .02 | 26 | .002 | .02 | 26 | .002 | .02 | 25 |
| Gender ego | .01 | .03 | 27 | .005 | .03 | 26 | .01 | .03 | 26 | .01 | .03 | 26 | .002 | .03 | 25 |
| Same gender | .35^***^ | .02 | 27 | .35^***^ | .02 | 26 | .35^***^ | .02 | 26 | .35^***^ | .02 | 26 | .35^***^ | .02 | 25 |
| Same ethnicity | .17^***^ | .02 | 27 | .18^***^ | .02 | 26 | .17^***^ | .02 | 26 | .18^***^ | .02 | 26 | .17^***^ | .02 | 25 |
| Academic drive alter | .05 | .01 | 27 | .05 | .01 | 26 | .05 | .01 | 26 | .05 | .01 | 26 | .05 | .01 | 25 |
| Academic drive ego | -.16^***^ | .02 | 27 | -.15^***^ | .02 | 26 | -.15^***^ | .02 | 26 | -.15^***^ | .02 | 26 | -.15^***^ | .02 | 25 |
| Similar academic drive | .01 | .10 | 27 | -.02 | .10 | 24 | .01 | .11 | 26 | .03 | .11 | 26 | .02 | .11 | 25 |
|  |  |  |  |  |  |  |  |  |  |  |  |  |  |  |  |

*Note.* Model 1 is idendical regardless of whether group membership is based on gender or ethnicity.

**Table 5**

Complete SAOM results – prejudice as an outcome and group membership based on ethnicity

|  | Model 1 | | | Model 2 | | | Model 3 | | | Model 4 | | | Model 5 | | |
| --- | --- | --- | --- | --- | --- | --- | --- | --- | --- | --- | --- | --- | --- | --- | --- |
|  | Estimate | SE | N | Estimate | SE | N | Estimate | SE | N | Estimate | SE | N | Estimate | SE | N |
| **Behavioral Function** |  |  |  |  |  |  |  |  |  |  |  |  |  |  |  |
| ***Normative pressure*** |  |  |  |  |  |  |  |  |  |  |  |  |  |  |  |
| Normative pressure  same-sex peers |  |  |  | 2.94^***^ | .58 | 26 | 1.10^***^ | .21 | 26 |  |  |  | .96^***^ | .22 | 25 |
| ***Friendship influence*** |  |  |  |  |  |  |  |  |  |  |  |  |  |  |  |
| Friendship influence | 1.01^***^ | .29 | 27 |  |  |  | 1.03^***^ | .30 | 26 |  |  |  |  |  |  |
| Friendship influence  same-gender peers |  |  |  |  |  |  |  |  |  | .69^**^ | .23 | 26 | .36 | .26 | 25 |
| Friendship influence  different-gender peers |  |  |  |  |  |  |  |  |  | .67^*^ | .29 | 26 | .81^**^ | .30 | 25 |
|  |  |  |  |  |  |  |  |  |  |  |  |  |  |  |  |
| ***Other effects*** |  |  |  |  |  |  |  |  |  |  |  |  |  |  |  |
| Rate 1 | 3.81^***^ | .27 | 27 | 3.85^***^ | .27 | 26 | 3.78^***^ | .28 | 26 | 3.80^***^ | .27 | 26 | 3.75^***^ | .28 | 25 |
| Rate 2 | 3.06^***^ | .25 | 27 | 3.10^***^ | .23 | 26 | 3.10^***^ | .24 | 26 | 3.03^***^ | .24 | 26 | 3.15^***^ | .25 | 25 |
| Linear shape | .22 | .38 | 26 | .37 | .27 | 26 | .50 | .35 | 26 | .39 | .32 | 26 | .53 | .33 | 25 |
| Quadratic shape | -.01 | .02 | 27 | .02 | .01 | 26 | .06 | .02 | 26 | -.01 | .02 | 26 | .06^**^ | .02 | 25 |
| School-grade average | -.16 | .21 | 27 | 1.13^***^ | .21 | 26 | -.26 | .21 | 25 | -.17 | .21 | 25 | -.27 | .20 | 23 |
| Being male vs. female | -.04 | .02 | 27 | -.05 | .02 | 26 | -.04 | .02 | 26 | -.04 | .03 | 26 | -.05 | .03 | 25 |
| **Network function** |  |  |  |  |  |  |  |  |  |  |  |  |  |  |  |
| Rate 1 | 12.63^***^ | .52 | 27 | 12.69^***^ | .54 | 26 | 12.69^***^ | .54 | 26 | 12.66^***^ | .54 | 26 | 12.65^***^ | .56 | 25 |
| Rate 2 | 12.02^***^ | .47 | 27 | 12.21^***^ | .46 | 26 | 12.20^***^ | .46 | 26 | 12.20^***^ | .46 | 26 | 12.16^***^ | .47 | 25 |
| Density | -1.85^***^ | .05 | 27 | -1.85^***^ | .06 | 26 | -1.85^***^ | .06 | 26 | -1.85^***^ | .06 | 26 | -1.87^***^ | .05 | 25 |
| Reciprocity | 1.66^***^ | .05 | 27 | 1.65^***^ | .05 | 26 | 1.65^***^ | .05 | 26 | 1.65^***^ | .05 | 26 | 1.65^***^ | .05 | 25 |
| GWESPFF | 1.92^***^ | .05 | 27 | 1.93^***^ | .05 | 26 | 1.93^***^ | .05 | 26 | 1.94^***^ | .05 | 26 | 1.93^***^ | .05 | 25 |
| GWESPBB | .69^***^ | .04 | 27 | .69^***^ | .04 | 26 | .69^***^ | .03 | 26 | .70^***^ | .04 | 26 | .70^***^ | .03 | 25 |
| GWESPRR | -1.02^***^ | .07 | 27 | -1.04^***^ | .07 | 26 | -1.04^***^ | .07 | 26 | -1.04^***^ | .07 | 26 | -1.04^***^ | .07 | 25 |
| Indegree popularity squared | -.17^***^ | .02 | 27 | -.18^***^ | .02 | 26 | -.17^***^ | .02 | 26 | -.17^***^ | .02 | 26 | -.16^***^ | .02 | 25 |
| Outdegree popularity squared | -.59^***^ | .02 | 27 | -.59^***^ | .02 | 26 | -.59^***^ | .02 | 26 | -.59^***^ | .02 | 26 | -.60^***^ | .03 | 25 |
| Gender alter | .005 | .03 | 27 | .002 | .02 | 26 | .003 | .02 | 26 | .003 | .02 | 26 | .004 | .02 | 25 |
| Gender ego | .01 | .03 | 27 | .007 | .03 | 26 | .01 | .03 | 26 | .01 | .03 | 26 | .005 | .03 | 25 |
| Same gender | .35^***^ | .02 | 27 | .35^***^ | .02 | 26 | .34^***^ | .02 | 26 | .35^***^ | .02 | 26 | .35^***^ | .02 | 25 |
| Same ethnicity | .17^***^ | .02 | 27 | .18^***^ | .02 | 26 | .18^***^ | .02 | 26 | .18^***^ | .02 | 26 | .18^***^ | .02 | 25 |
| Academic drive alter | .05 | .01 | 27 | .04 | .01 | 26 | .05 | .01 | 26 | .05^***^ | .01 | 26 | .05 | .01 | 25 |
| Academic drive ego | -.16^***^ | .02 | 27 | -.15^***^ | .02 | 26 | -.15^***^ | .02 | 26 | -.15^***^ | .02 | 26 | -.15^***^ | .02 | 25 |
| Similar academic drive | .01 | .10 | 27 | .004 | .11 | 24 | .01 | .11 | 26 | .01 | .11 | 26 | .02 | .11 | 25 |
|  |  |  |  |  |  |  |  |  |  |  |  |  |  |  |  |

*Note.* Model 1 is idendical regardless of whether group membership is based on gender or ethnicity.

**Table 6**

Complete SAOM results – religiosity as an outcome and group membership based on gender

|  | Model 1 | | | Model 2 | | | Model 3 | | | Model 4 | | | Model 5 | | |
| --- | --- | --- | --- | --- | --- | --- | --- | --- | --- | --- | --- | --- | --- | --- | --- |
|  | Estimate | SE | N | Estimate | SE | N | Estimate | SE | N | Estimate | SE | N | Estimate | SE | N |
| **Behavioral Function** |  |  |  |  |  |  |  |  |  |  |  |  |  |  |  |
| ***Normative pressure*** |  |  |  |  |  |  |  |  |  |  |  |  |  |  |  |
| Normative pressure  same-sex peers |  |  |  | -5.16^***^ | 1.45 | 29 | -8.57^***^ | .1.60 | 28 |  |  |  | -.9.02^***^ | 1.81 | 26 |
| ***Friendship influence*** |  |  |  |  |  |  |  |  |  |  |  |  |  |  |  |
| Friendship influence | 2.55^***^ | .29 | 29 |  |  |  | 2.82^***^ | .30 | 28 |  |  |  |  |  |  |
| Friendship influence  same-gender peers |  |  |  |  |  |  |  |  |  | 2.11^***^ | .30 | 27 | 2.46 ^***^ | .33 | 26 |
| Friendship influence  different-gender peers |  |  |  |  |  |  |  |  |  | -.07 | .50 | 26 | -.16 | .53 | 23 |
|  |  |  |  |  |  |  |  |  |  |  |  |  |  |  |  |
| ***Other effects*** |  |  |  |  |  |  |  |  |  |  |  |  |  |  |  |
| Rate 1 | 3.47^***^ | .32 | 29 | 3.74^***^ | .34 | 29 | 3.53^***^ | .33 | 28 | 3.49^***^ | .35 | 27 | 3.47^***^ | .34 | 26 |
| Rate 2 | 3.21^***^ | .24 | 29 | 3.46^***^ | .26 | 29 | 3.31^***^ | .24 | 28 | 3.14^***^ | .25 | 27 | 3.29^***^ | .26 | 26 |
| Linear shape | -.31^***^ | .07 | 29 | -1.38^***^ | .34 | 29 | -1.32^***^ | .30 | 28 | -.40^**^ | .12 | 27 | -1.54^*^ | .31 | 26 |
| Quadratic shape | .23 | .02 | 29 | -.15^*^ | .06 | 29 | -.16^*^ | .06 | 28 | .22^***^ | .02 | 27 | -.19^*^ | .07 | 26 |
| School-grade average | -.12 | .22 | 29 | 1.02^**^ | .32 | 29 | .99^**^ | .35 | 28 | -.07 | .24 | 27 | 1.06^**^ | .38 | 26 |
| Being male vs. female | -.03 | .03 | 29 | -.05 | .04 | 29 | -.07 | .05 | 28 | -.005 | .03 | 27 | -.07 | .06 | 26 |
| **Network function** |  |  |  |  |  |  |  |  |  |  |  |  |  |  |  |
| Rate 1 | 12.79^***^ | .56 | 29 | 12.76^***^ | .56 | 29 | 12.97^***^ | .55 | 28 | 12.64^***^ | .57 | 27 | 12.96^***^ | .59 | 26 |
| Rate 2 | 12.24^***^ | .47 | 29 | 12.20^***^ | .47 | 29 | 12.20^***^ | .48 | 28 | 12.26^***^ | .50 | 27 | 12.32^***^ | .52 | 26 |
| Density | -1.78^***^ | .06 | 29 | -1.78^***^ | .06 | 29 | -1.79^***^ | .06 | 28 | -1.77^***^ | .07 | 27 | -1.77^***^ | .07 | 26 |
| Reciprocity | 1.63^***^ | .04 | 29 | 1.63^***^ | .04 | 29 | 1.63^***^ | .04 | 28 | 1.63^***^ | .05 | 27 | 1.63^***^ | .05 | 26 |
| GWESPFF | 1.89^***^ | .05 | 29 | 1.89^***^ | .05 | 29 | 1.90^***^ | .05 | 28 | 1.87^***^ | .05 | 27 | 1.89^***^ | .05 | 26 |
| GWESPBB | .69^***^ | .03 | 29 | .69^***^ | .03 | 29 | .69^***^ | .03 | 28 | .67^***^ | .03 | 27 | .69^***^ | .03 | 26 |
| GWESPRR | -1.00^***^ | .06 | 29 | -1.00^***^ | .06 | 29 | -1.00^***^ | .06 | 28 | -.97^***^ | .07 | 27 | -.98^***^ | .07 | 26 |
| Indegree popularity squared | -.19^***^ | .02 | 29 | -.19^***^ | .02 | 29 | -.19^***^ | .02 | 28 | -.18^***^ | .02 | 27 | -.18^***^ | .02 | 26 |
| Outdegree popularity squared | -.61^***^ | .02 | 29 | -.61^***^ | .02 | 29 | -.61^***^ | .02 | 28 | -.61^***^ | .03 | 27 | -.61^***^ | .02 | 26 |
| Gender alter | .01 | .02 | 29 | .01 | .02 | 29 | .01 | .02 | 28 | .02 | .02 | 27 | .02 | .02 | 26 |
| Gender ego | .03 | .03 | 29 | .03 | .03 | 29 | .03 | .03 | 28 | .03 | .03 | 27 | .02 | .03 | 26 |
| Same gender | .35^***^ | .02 | 29 | .35^***^ | .02 | 29 | .35^***^ | .02 | 28 | .36^***^ | .02 | 27 | .35^***^ | .02 | 26 |
| Same ethnicity | .17^***^ | .02 | 29 | .17^***^ | .02 | 29 | .17^***^ | .02 | 28 | .17^***^ | .02 | 27 | .16^***^ | .03 | 26 |
| Academic drive alter | .04^***^ | .01 | 29 | .04 | .01 | 29 | .04^***^ | .01 | 28 | .04^***^ | .01 | 27 | .04 | .01 | 26 |
| Academic drive ego | -.12^***^ | .02 | 29 | -.12^***^ | .02 | 29 | -.12^***^ | .02 | 28 | -.12^***^ | .02 | 27 | -.12^***^ | .02 | 26 |
| Similar academic drive | .13^*^ | .06 | 29 | .13^*^ | .06 | 29 | .13^*^ | .06 | 28 | .10 | .07 | 27 | .09 | .07 | 26 |
|  |  |  |  |  |  |  |  |  |  |  |  | 27 |  |  |  |

*Note.* Model 1 is idendical regardless of whether group membership is based on gender or ethnicity.

**Table 7**

Complete SAOM results – religiosity as an outcome and group membership based on ethnicity

|  | Model 1 | | | Model 2 | | | Model 3 | | | Model 4 | | | Model 5 | | |
| --- | --- | --- | --- | --- | --- | --- | --- | --- | --- | --- | --- | --- | --- | --- | --- |
|  | Estimate | SE | N | Estimate | SE | N | Estimate | SE | N | Estimate | SE | N | Estimate | SE | N |
| **Behavioral Function** |  |  |  |  |  |  |  |  |  |  |  |  |  |  |  |
| ***Normative pressure*** |  |  |  |  |  |  |  |  |  |  |  |  |  |  |  |
| Normative pressure  same-sex peers |  |  |  | 1.06^***^ | .16 | 29 | .92^***^ | .17 | 29 |  |  |  | .76 ^***^ | .20 | 28 |
| ***Friendship influence*** |  |  |  |  |  |  |  |  |  |  |  |  |  |  |  |
| Friendship influence | 2.55^***^ | .29 | 29 |  |  |  | 2.37^***^ | .29 | 29 |  |  |  |  |  |  |
| Friendship influence  same-gender peers |  |  |  |  |  |  |  |  |  | 1.38^***^ | .22 | 29 | 1.22^***^ | .25 | 28 |
| Friendship influence  different-gender peers |  |  |  |  |  |  |  |  |  | 1.41^***^ | .29 | 29 | 1.41^***^ | .31 | 28 |
|  |  |  |  |  |  |  |  |  |  |  |  |  |  |  |  |
| ***Other effects*** |  |  |  |  |  |  |  |  |  |  |  |  |  |  |  |
| Rate 1 | 3.47^***^ | .32 | 29 | 3.63^***^ | .34 | 29 | 3.54^***^ | .34 | 29 | 3.48^***^ | .33 | 29 | 3.49^***^ | .33 | 28 |
| Rate 2 | 3.21^***^ | .24 | 29 | 3.32^***^ | .25 | 29 | 3.27^***^ | .25 | 29 | 3.18^***^ | .25 | 29 | 3.18^***^ | .25 | 28 |
| Linear shape | -.31^***^ | .07 | 29 | -.35^***^ | .10 | 29 | -.27^***^ | .06 | 29 | -.43^**^ | .12 | 29 | -.26^***^ | .07 | 28 |
| Quadratic shape | .23 | .02 | 29 | .18^***^ | .02 | 29 | .25^***^ | .02 | 29 | .22^***^ | .02 | 29 | .25^***^ | .03 | 28 |
| School-grade average | -.12 | .22 | 29 | .08 | .20 | 29 | -.19 | .23 | 27 | -.04 | .22 | 26 | -.06 | .24 | 26 |
| Being male vs. female | -.03 | .03 | 29 | -.02 | .03 | 29 | -.03 | .03 | 29 | -.02 | .03 | 29 | -.02 | .03 | 28 |
| **Network function** |  |  |  |  |  |  |  |  |  |  |  |  |  |  |  |
| Rate 1 | 12.79^***^ | .56 | 29 | 12.79^***^ | .56 | 29 | 12.79^***^ | .56 | 29 | 12.79^***^ | .56 | 29 | 12.85^***^ | .58 | 28 |
| Rate 2 | 12.24^***^ | .47 | 29 | 12.19^***^ | .47 | 29 | 12.23^***^ | .48 | 29 | 12.20^***^ | .47 | 29 | 12.12^***^ | .47 | 28 |
| Density | -1.78^***^ | .06 | 29 | -1.78^***^ | .06 | 29 | -1.79^***^ | .06 | 29 | -1.78^***^ | .06 | 29 | -1.79^***^ | .06 | 28 |
| Reciprocity | 1.63^***^ | .04 | 29 | 1.63^***^ | .04 | 29 | 1.63^***^ | .04 | 29 | 1.63^***^ | .04 | 29 | 1.64^***^ | .04 | 28 |
| GWESPFF | 1.89^***^ | .05 | 29 | 1.89^***^ | .05 | 29 | 1.89^***^ | .05 | 29 | 1.89^***^ | .05 | 29 | 1.91^***^ | .04 | 28 |
| GWESPBB | .69^***^ | .03 | 29 | .68^***^ | .03 | 29 | .68^***^ | .03 | 29 | .69^***^ | .03 | 29 | .69^***^ | .03 | 28 |
| GWESPRR | -1.00^***^ | .06 | 29 | -.99^***^ | .06 | 29 | -1.00^***^ | .06 | 29 | -1.00^***^ | .06 | 29 | -1.02^***^ | .06 | 28 |
| Indegree popularity squared | -.19^***^ | .02 | 29 | -.19^***^ | .02 | 29 | -.19^***^ | .02 | 29 | -.19^***^ | .02 | 29 | -.20^***^ | .02 | 28 |
| Outdegree popularity squared | -.61^***^ | .02 | 29 | -.61^***^ | .02 | 29 | -.60^***^ | .02 | 29 | -.61^***^ | .03 | 29 | -.60^***^ | .02 | 28 |
| Gender alter | .01 | .02 | 29 | .01 | .02 | 29 | .01 | .02 | 29 | .01 | .02 | 29 | .01 | .02 | 28 |
| Gender ego | .03 | .03 | 29 | .03 | .03 | 29 | .03 | .03 | 29 | .03 | .03 | 29 | .03 | .03 | 28 |
| Same gender | .35^***^ | .02 | 29 | .35^***^ | .02 | 29 | .35^***^ | .02 | 29 | .35^***^ | .02 | 29 | .35^***^ | .02 | 28 |
| Same ethnicity | .17^***^ | .02 | 29 | .17^***^ | .02 | 29 | .17^***^ | .02 | 29 | .17^***^ | .02 | 29 | .17^***^ | .02 | 28 |
| Academic drive alter | .04^***^ | .01 | 29 | .04 | .01 | 29 | .04^***^ | .01 | 29 | .04^***^ | .01 | 29 | .04 | .01 | 28 |
| Academic drive ego | -.12^***^ | .02 | 29 | -.11^***^ | .02 | 29 | -.12^***^ | .02 | 29 | -.12^***^ | .02 | 29 | -.12^***^ | .02 | 28 |
| Similar academic drive | .13^*^ | .06 | 29 | .14^*^ | .06 | 29 | .13^*^ | .06 | 29 | .14^*^ | .06 | 29 | .13^*^ | .06 | 28 |
|  |  |  |  |  |  |  |  |  |  |  |  |  |  |  |  |

*Note.* Model 1 is idendical regardless of whether group membership is based on gender or ethnicity.

**Heterogeneity tests of key effects**

We investigated whether the key effects reported in Figure 5 in the manuscript—friendship influence, normative pressure, and influence of ingroup and outgroup friends—significantly varied across school-grades. For normative pressure and friendship influence, we provide a test for residual heterogeneity based on Model 3. The heterogeneity test for the influence of ingroup and outgroup friends is based on Model 5.

Normative pressure significantly varied only in the case of academic drive (gender: *Q*(27) = 41.26, *p* = .039; ethnicity: *Q*(28) = 18.36, *p* = .917). It did not vary in the case of prejudice (*Q*(25) = 24.94, *p* = 0.466; ethnicity: *Q*(25) = 24.94, *p* = 0.466), and religiosity (gender: *Q*(27) = 31.98, *p* = 0.233; ethnicity: *Q*(28) = 34.64, *p* = 0.181). Friendship influence did not significantly vary in the case of academic drive (gender: *Q*(27) = 17.32, *p* = 0.923; ethnicity: *Q*(28) = 17.7402, *p* = .933), prejudice (gender: *Q*(25) = 12.63, *p* = 0.981; ethnicity: *Q*(25) = 10.88, *p* = 0.994), and religiosity (*gender: Q*(27) = 20.41, *p* = 0.813; ethnicity: *Q*(28) = 20.70, *p* = 0.838).

**Table 8**

*Deviations from the preregistration*

| Changes in | Preregistered | Deviation |
| --- | --- | --- |
| Baseline model | Estimation of a baseline model and five models for hypothesis testing. | The baseline model was not reported, as it did not contribute substantively to hypothesis testing. Instead, we reported the five preregistered models that were central to addressing our research questions. |
| Missing values approach | No approach to handling missing values was preregistered. | We addressed missing data using multiple imputation. |
| Simulations | We preregistered simulations to further test the role of normative pressure. | We did not conduct the simulations, as the role of normative pressure in shaping the outcomes was sufficiently supported by the empirical models alone. |
